# Ancestry specific associations of a genetic risk score, dietary patterns and metabolic syndrome: a longitudinal ARIC study

**DOI:** 10.1186/s12920-021-00961-8

**Published:** 2021-05-01

**Authors:** Dale S. Hardy, Susan B. Racette, Jane T. Garvin, Hirut T. Gebrekristos, Tesfaye B. Mersha

**Affiliations:** 1 Department of Internal Medicine, Morehouse School of Medicine, 720 Westview Drive, Atlanta, GA 30310 USA; 2 Program in Physical Therapy and Department of Medicine, Washington University School of Medicine, St. Louis, MO 63108 USA; 3 College of Nursing, Augusta University, Augusta, GA 30912 USA; 4 Division of Asthma Research, Department of Pediatrics, Cincinnati Children’s Hospital Medical Center, University of Cincinnati, 3333 Burnet Ave, Cincinnati, OH 45229 USA

**Keywords:** Genetic risk score, Polymorphism, Dietary patterns, Interaction, Metabolic syndrome, Race, Ancestry

## Abstract

**Background:**

Associations have been observed among genetic variants, dietary patterns, and metabolic syndrome (MetS). A gap in knowledge is whether a genetic risk score (GRS) and dietary patterns interact to increase MetS risk among African Americans. We investigated whether MetS risk was influenced by interaction between a GRS and dietary patterns among Whites and African Americans. A secondary aim examined if molecular genetic clusterings differed by racial ancestry.

**Methods:**

We used longitudinal data over 4-visits (1987–1998) that included 10,681 participants aged 45–64y at baseline from the Atherosclerosis Risk in Communities study (8451 Whites and 2230 African Americans). We constructed a simple-count GRS as the linear weighted sum of high-risk alleles (0, 1, 2) from cardiovascular disease polymorphisms from the genome-wide association studies catalog associated with MetS risk. Three dietary patterns were determined by factor analysis of food frequency questionnaire data: Western, healthy, and high-fat dairy. MetS was defined according to the 2016 National Cholesterol Education Program Adult Treatment Panel III criteria but used 2017 American Heart Association/American College of Cardiology criteria for elevated blood pressure. Analyses included generalized linear model risk ratios (RR), 95% confidence intervals (CI), and Bonferroni correction for multiple testing.

**Results:**

The Western dietary pattern was associated with higher risk for MetS across increasing GRS tertiles among Whites (*p* < 0.017). The high-fat dairy pattern was protective against MetS, but its impact was most effective in the lowest GRS tertile in Whites (RR = 0.62; CI: 0.52–0.74) and African Americans (RR = 0.67; CI: 0.49–0.91). Among each racial group within GRS tertiles, the Western dietary pattern was associated with development and cycling of MetS status between visits, and the high-fat dairy pattern with being free from MetS (*p* < 0.017). The healthy dietary pattern was associated with higher risk of MetS among African Americans which may be explained by higher sucrose intake (*p* < 0.0001). Fewer genes, but more metabolic pathways for obesity, body fat distribution, and lipid and carbohydrate metabolism were identified in African Americans than Whites. Some polymorphisms were linked to the Western and high-fat dairy patterns.

**Conclusion:**

The influence of dietary patterns on MetS risk appears to differ by genetic predisposition and racial ancestry.

**Supplementary Information:**

The online version contains supplementary material available at 10.1186/s12920-021-00961-8.

## Background

Metabolic syndrome (MetS) is a condition that approximately doubles the risk of incident type 2 diabetes and cardiovascular disease [1, 2], and increases mortality risk over fourfold [3]. MetS is characterized by clusters of ≥ 3 metabolic traits as defined by National Cholesterol Education Program Adult Treatment Panel III criteria: abnormal fasting blood glucose levels, central obesity, dyslipidemia, and elevated blood pressure [4]. Higher body mass index (BMI) and current cigarette smoking are associated with higher risk for MetS [5]. The prevalence of MetS is higher in older adults and in those with low levels of physical activity [6]. The etiology of MetS is complex, but reports have shown that genetic polymorphisms [7–12], dietary patterns [13–17], and physical activity play a role in its pathogenesis.

Genetic risk scores (GRSs) composed of single nucleotide polymorphisms (SNPs) have been associated with higher risk of MetS and cardiovascular diseases. A GRS was associated with MetS risk, type 2 diabetes, obesity, and cardiovascular diseases in several studies [18–30]. A GRS was associated with MetS risk in Koreans, but more in Korean females than Korean males [18]. GRSs for type 2 diabetes were associated with a Western dietary pattern in men [19], and mediated 9% risk of type 2 diabetes between parent and offspring [20]. Other GRSs for obesity risk were associated with BMI and obesity in European ancestry (Whites) and African Americans [21], predicted posttraumatic stress disorder-related MetS [22], and increased MetS risk in Chinese children [23]. Another study showed that a GRS composed of coronary artery disease SNPs was associated with not only coronary heart disease, but stroke, peripheral vascular disease, heart failure, and atrial fibrillation [24]. There is evidence that polymorphisms from the FTO alpha-ketoglutarate dependent dioxygenase (renamed from fat mass and obesity-associated FTO): rs9939609, rs8050135, and rs1420185 are associated with higher risk for MetS [31–33].

Various mechanisms have been proposed regarding the influence of dietary patterns on development of MetS, but these mechanisms have not been fully elucidated. Evidence from observational studies suggests that meat, fried foods, and diet soda increase the risk of incident MetS [8, 9, 34, 35], whereas higher intakes of fruits, vegetables, and whole grains have protective associations with MetS and its components [7, 11, 36]. Dietary patterns provide a comprehensive depiction of typical eating habits which may be more informative than analyses of individual foods. Most importantly, studies have shown that Western dietary patterns that were comprised of fried foods, soda, meat, and alcohol were associated with MetS risk [13–17].

Studies have reported interactions between a GRS, SNPs and dietary patterns. Qi et al. [19] reported that a Western dietary pattern increased the risk of type 2 diabetes and the highest risk was in the highest GRS tertile. Other studies reported that a GRS and a diet score increased the risk for obesity in Whites [37]. Interaction was present for rs10738760, a SNP of Vascular Endothelial Growth Factor, and high fat and sugar intake to increase risk for MetS [38]. Other interactions were reported for FTO rs9939609 with protein and carbohydrate intake on BMI [39]. Other studies reported that homozygous carriers for FTO SNPs: rs9939609, rs17817449, rs8050136 were associated with carbohydrate and protein intake in Whites and African Americans and rs8050136 with fat intake in Whites [40]. In addition, Angiopoietin-like 4 protein (ANGPTL4) interacted with carbohydrate intake to increase HDL cholesterol levels [41, 42].

Although MetS is influenced by genetics and dietary patterns, the potential pathways linking them have not been examined systematically in a population-based study. In this study, we investigated whether a GRS interacted with dietary patterns to increase MetS risk among Whites and African Americans followed over 11 years. A secondary aim was to investigate whether the molecular genetic clusterings associated with MetS differed by racial ancestry.

## Methods

### Study population

Atherosclerosis Risk in Communities (ARIC) study data at baseline (1987–1989) and three follow-up visits (1990–1992, 1993–1995, and 1996–1998) were obtained from the database of Genotypes and Phenotypes (dbGaP) [43]. The ARIC study, sponsored by the National Heart, Lung, and Blood Institute, is a large-scale, ongoing prospective cohort study conducted in four U.S. communities: Jackson, MS; Forsyth County, NC; Minneapolis, MN; and Washington County, MD. ARIC was designed to investigate the etiology and natural history of atherosclerosis, as well as the etiology of clinical atherosclerotic diseases and their sequelae. The overall participation rate was 60%, including 42% and 49% of eligible African American men and women, respectively, and 67% and 68% of eligible White men and women respectively, who were 45 to 64 years old [44]. All participants signed an informed consent document. Further design and sampling methods are explained elsewhere [45]. The current study was approved by the Social & Behavioral Institutional Review Board at Morehouse School of Medicine.

### Inclusion of participants into the study

**Fig. 1:**
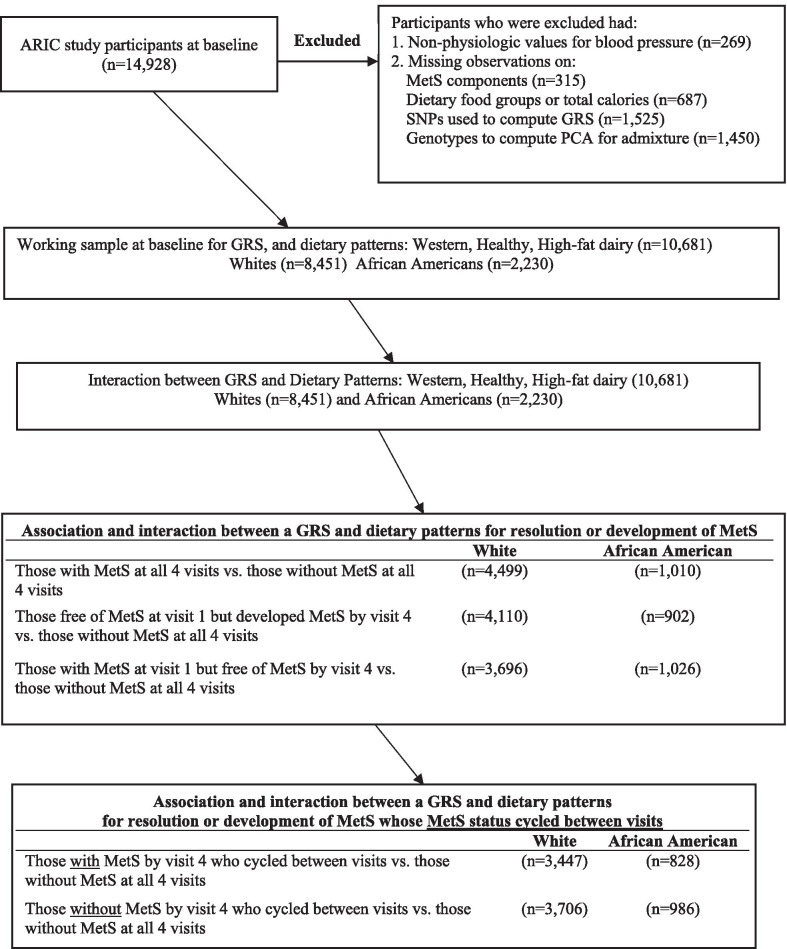
Entry of participants into the study and partitioned for analysis by racial ancestry. ARIC, Atherosclerosis Risk in Communities; MetS, metabolic syndrome; GRS, genetic risk score; PCA, principal components analysis

The original ARIC sample included 14,928 participants at baseline. We imputed missing observations on cigarette smoking, drinking status, sports physical activity and education level to augment our sample, especially for African Americans. Imputations were < 2% of the original participant sample. Figure 1 below depicts how participants were allocated into the study. To assimilate a sample population with physiologic blood pressure values, participants were excluded from the analysis at baseline and follow-up if they had systolic blood pressure < 80 mmHg (n = 54), diastolic blood pressure < 45 mmHg (n = 168), a difference of < 20 mmHg between systolic and diastolic blood pressures (n = 23), or mean arterial pressure < 60 mmHg (n = 24). Additional participants were excluded if they had missing observations at baseline for MetS component risk factors (n = 315), dietary food groups or total calories (n = 687), SNPs (n = 1526), or 20 genetic principal components to correct for population admixture (n = 1450). After excluding individuals with missing observations on food variables at baseline, there were no observations that had caloric intake < 600 or > 4200 kcal/day for men, and < 500 or > 3600 kcal/day for women. Our final models at baseline included 10,681 participants, of which 8451 (79.1%) were White and 2230 (20.9%) were African American.

### Food frequency questionnaire

A 66-item semi-quantitative food frequency questionnaire (FFQ) was used to obtain information on dietary intake. The FFQ was a modified version of the 61-item FFQ developed and validated by Willett et al. [46]. At the examination interview, participants were shown standard serving sizes and typical servings using food models to help them estimate intake. Participants reported their intake based on 9 levels of frequency, ranging from < 1 time per month to ≥ 6 times per day and were asked additional information such as brand names. The FFQ was administered during the first study visit (1987–1989) and the third study visit (1993–1995); a sub-sample of participants also completed the FFQ at the second visit (n = 1004). For participants without FFQ data at visit 2, we carried forward FFQ visit 1 data. Likewise, because the FFQ was not administered at ARIC visit 4, visit 3 FFQ data were carried forward to visit 4. The justification for this approach is that studies have shown that there is little change in dietary and physical activity patterns over a short-term, even after a cardiovascular event [55, 56].

### Dietary patterns by racial ancestry

From our FFQ data, three dietary patterns emerged based on factor analysis, with the principal components factor option, varimax rotation, and a correlation ≥ 0.3: Western, healthy, and high-fat dairy. Briefly, we conducted factor analysis from 39 food or food groups based on 66 predefined foods or food groups from ARIC FFQs by racial ancestry. The dietary pattern scores were calculated by summing the standardized intakes of the component foods, weighted by the factor loadings of the foods. The dietary pattern scores rank participants according to the degree to which they adhere to the corresponding dietary patterns. Additional file 1: Table S1 shows the food groups used to form the dietary patterns, and Additional file 1: Table S2 shows the food groups and their factor loadings ≥ 0.3 by racial ancestry. Dietary patterns were chosen based on how the foods clustered together (Additional file 1: Table S2). A Western dietary pattern among Whites was characterized by red and processed meat, chicken with skin, and fried foods; among African Americans, processed meat and fried foods were more prevalent. A healthy dietary pattern consisted of rice, pasta, vegetables, chicken without skin, and lentils/ beans among Whites; among African Americans, there was a greater variety of foods. The high-fat dairy pattern included whole milk and butter and was similar for both racial ancestry groups. The proportions of total carbohydrate, protein and fat intake as a percentage of calories were not significantly different among Whites and African Americans. However, the median sucrose intake (g) differed between the racial ancestry groups (Whites: 39.2% vs. African Americans: 45.8%; *p* < 0.0001).

From our factor analysis, we labelled the first, second and seventh factors for Whites and the fourth, second, and ninth factors for African Americans as Western, healthy and high-fat dairy patterns, respectively. The variance/percent variance explained for the respective patterns were 2.6/6.5%, 2.0/5.0%, and 1.4/3.5% for Whites and 1.8/4.6%, 2.9/7.3%, and 1.4/4.5% for African Americans.

### DNA extraction and genotyping

Genotyping was performed on whole blood using the TaqMan assay (Applied Biosystems, Foster City, CA, USA), the Affymetrix 6.0 single nucleotide polymorphism array, and the Birdseed calling algorithm [47] at the Broad Institute Center for Genotyping and Analysis. Allele detection was carried-out using the ABI Prism 7700 Sequence Detection System (Applied Biosystems).

### Construction of the GRS

We compiled 16,495 SNPs chosen from preselected reference risk alleles associated with MetS, its components, and cardiovascular disease from the NHGRI GWAS catalog to compute a risk-raising allele GRS by racial ancestry [1, 48, 49]. The ARIC GWAS data yielded 397 SNPs. We then regressed MetS against each of the 397 SNPs, adjusted for age and sex by racial ancestry. SNPs with *p* value < 0.1 were chosen to be included in the GRS. We calculated the GRS using a simple count method as a variable whose values were equal to the number of copies of the high-risk allele under the additive model (0, 1, 2). Cardiovascular diseases searched in the NHGRI GWAS catalog were ‘metabolic syndrome, components of MetS and related phenotypes (glucose, triglycerides, high blood pressure, waist circumference, BMI, HDL cholesterol, lipids), diabetes, type 2 diabetes, stroke, coronary heart disease, peripheral artery or vascular disease, insulin resistance, inflammation, and psychological disorders linked with excessive food intake (gorging, bipolar disorder, bulimia). Additional file 1: Tables S3A and S3B show the chromosomes, SNPs, and genes used in the GRSs by racial ancestry. Most SNPs mapped to genes with lipid phenotypes such as total cholesterol, triglycerides, and HDL cholesterol.

For SNPs within high linkage disequilibrium ≥ 0.8, tag SNPs were chosen based on higher binding capacity [50]. The Hardy–Weinberg test for all SNPs was performed in Plink [49] using chi-square goodness-of-fit test for Whites and African Americans separately. All SNPs chosen for the GRS for Whites and African Americans were found to be in Hardy–Weinberg equilibrium (*p* > 0.05). The 20 genetic principal components were computed in Linux using gcta64 guidelines [51] to calculate a genetic-related matrix by racial ancestry and then specifying the principal components.

### Study variables

The primary outcome was MetS, defined according to the National Cholesterol Education Program Adult Treatment Panel III criteria, as clusters of ≥ 3 cardiovascular disease risk factors that include abdominal obesity based on waist circumference (men: > 102 cm, women: > 88 cm), elevated fasting blood glucose (≥ 100 mg/dL), elevated blood pressure based on the 2017 guidelines of the American Heart Association/American College of Cardiology (AHA/ACC) (≥ 120 mmHg systolic or ≥ 80 mmHg diastolic or taking blood pressure medications) [52], low HDL cholesterol (men: < 40 mg/dL, women: < 50 mg/dL), and high triglycerides (≥ 150 mg/dL) [1, 53, 54]. The main predictor variable was the GRS that was constructed using values equal to the number of copies of the high-risk allele under the additive model (0, 1, 2).

The interaction variables were the GRS and dietary patterns (described in detail above). The stratification variable was racial ancestry, defined as White or African American based on self-report. The following participant characteristics were considered for covariate adjustment to create a covariate summary score: age (continuous), gender, educational status at visit 1, cigarette smoking status (never/ former/ current), alcohol drinking status (never/ former/ current), physical activity from the Baecke questionnaire (sports physical activity, a continuous variable) [57], and study visit, an ordinal variable (1 to 4) that depicts time in the study. As with the FFQ, because physical activity was collected at visits 1 and 3 only, we carried forward physical activity Baecke scores from visit 1 to visit 2, and from visit 3 to visit 4. We did not include BMI in covariate adjustment because BMI is in the causal pathway of the dietary patterns and MetS outcome.

### Statistical analysis

We regressed MetS against each SNP by racial ancestry (White or African American). SNPs with a *p* value < 0.1 were chosen for inclusion in the GRS. We computed a covariate summary score by regressing MetS on the covariates (age, gender, physical activity, education level at visit 1, cigarette smoking status, drinking status, study visit, and 20 genetic principal components for admixture) and then predicted the residuals. Analyses were performed using generalized linear model with Poisson family and log link to derive risk ratios (RR) and 95% confidence intervals (CI), after applying within-subject identification to specify responses that were nested in the participant in order to compute the cumulative effect of the GRS and/or dietary pattern over time. In our generalized linear model analysis, we were interested in the expectation of the outcome, MetS, as a function of the GRS and/or the dietary pattern adjusted for the covariate summary score. In our multivariate models, we regressed MetS on the GRS and/or each dietary pattern, adjusting for the covariate summary score.

We tested whether there was additive interaction (biological interaction) between the GRS and dietary patterns on the additive risk scale. In additive interaction models, we included the GRS, dietary pattern score, interaction term composed of the GRS and the dietary pattern, along with the covariate summary score for adjustment. To determine whether there was additive interaction, the GRS, dietary pattern, and their interaction term all had to be statistically significant at *p* < 0.05 using the Wald test [58]. All analyses were reported as per 5-increment increase in dietary pattern scores by GRS tertiles. In all models, a 2-sided *p* < 0.05 and more stringent, Bonferroni adjustment for multiple testing of *p* < 0.017 were used as the threshold for statistical significance in the final models. All regression analyses were bootstrapped 10,000 times. Our statistical analyses were conducted using Stata MP, version 16.0 (StataCorp, College Station, TX).

### Literature Lab™-based clustering

A secondary objective of our study was to investigate the biological relationships among the genes linked to the SNPs by racial ancestry using Literature Lab™ from Acumenta Biotech Clustering algorithm [59]. In Literature Lab™, clustering analysis is performed on the unique qualified terms (Strong, Moderate, or Positive) from domains identified in the analysis of the gene list using hierarchical clustering with uncentered correlation and average linkage, software package Cluster 3.0 (http://bonsai.hgc.jp/~mdehoon/software/cluster/software.htm). Genes were clustered on similarity in molecular function among each other.

Likewise, terms are clustered by how similarly they are related to the genes (percent of match that mention a given term and gene in PubMed). Clusters of ≥ 6 genes were then examined in decreasing order of the average intensity of the measure (percentage of gene abstracts mentioning each term). Multiple term clusters for the same gene cluster and multiple gene clusters for the same term cluster are shown together in the list of clusters and in the heatmap displays for the clusters. The selected clusters are presented both in a list of the clusters with terms and genes and in individual heat maps for each cluster. In the heat maps, yellow is used to signify intensity; the brightest yellow represents an intensity measure of 25% or more. An intensity of 0% is shown in black and intermediate intensities are shown in various shades from black to yellow.

## Results

### Descriptive characteristics by racial ancestry

At baseline (1987–1989), the total sample consisted of 10,681 participants (8451 (79.1%) White and 2230 (20.0%) African American) aged 45–64 years. Included in the total sample, MetS cases at baseline and during 11-years of follow-up included 43.0% and 50.5% among Whites and 49.2% and 55.8% among African Americans, respectively.

Table 1 shows the baseline characteristics of participants included in the analyses. All characteristics were significantly different between Whites and African Americans except total cholesterol and LDL cholesterol. A higher proportion of African Americans smoked cigarettes, had a high-school education or less, were on blood pressure medications or were obese; additionally, they had lower levels of physical activity, higher systolic and diastolic blood pressure, higher fasting blood glucose, and higher triglyceride levels compared to Whites. In contrast, a higher proportion of Whites were current drinkers and the mean HDL cholesterol was lower, but fewer had diabetes or MetS compared to African Americans.

**Table 1:** Characteristics among Whites and African Americans at baseline (1987–1989) in ARIC

	All races combined	Whites	African Americans	*P* value comparing African Americans to Whites
	(n = 10,681)	(n = 8451)	(n = 2230)	
Characteristic	Mean (SD) or column percent (%) of participants			
Age (45–64 years)	54.2 (5.6)	54.4 (5.7)	53.6 (5.8)	< 0.0001
Female (%)	54.9	53.0	62.0	< 0.0001
Body mass index (BMI)	27.6 (5.3)	27.0 (4.8)	29.7 (6.1)	< 0.0001
Weight status (%)				< 0.0001
Underweight (BMI ≤ 18.5 kg/m^2^)	0.8	0.8	0.8	
Normal weight (BMI 18.5–24.9 kg/m^2^)	33.0	36.4	20.9	
Overweight (BMI 25.0–29.9 kg/m^2^)	39.5	40.1	37.4	
Obese (BMI ≥ 30 kg/m^2^)	26.7	22.7	41.0	
Physical activity level (1–5 Baecke units)	2.5 (.8)	2.5 (0.8)	2.1 (0.7)	< 0.0001
Cigarette smoking (%)				< 0.0001
Current	25.1	24.1	28.9	
Former	33.1	35.7	24.1	
Never	41.7	40.1	47.0	
Alcohol intake (%)				< 0.0001
Current	58.1	65.4	32.1	
Former	17.9	16.6	22.5	
Never	24.0	17.9	44.4	
Education level (%)				< 0.0001
Grade school or zero years of education	8.5	5.6	18.7	
High school, but no degree	13.3	11.0	21.4	
High school graduate	33.1	36.3	21.5	
Vocational school	8.7	9.2	6.9	
College	26.7	29.1	18.2	
Graduate or professional school	9.9	8.9	13.4	
Systolic blood pressure (mmHg)	120.9 (18.1)	118.8 (16.8)	128.5 (20.4)	< 0.0001
Diastolic blood pressure (mmHg)	73.6 (10.7)	71.8 (9.7)	79.8 (11.9)	< 0.0001
Blood pressure medications (%)	29.7	25.5	44.7	< 0.0001
Fasting blood glucose (mg/dL)	108.1 (38.4)	105.3 (30.9)	117.8 (56.6)	< 0.0001
Diabetes (%)	11.1	8.7	19.4	< 0.0001
Metabolic syndrome (%)	44.3	43.0	49.2	< 0.0001
Waist circumference (cm)	96.9 (13.7)	96.2 (13.2)	99.3 (15.1)	< 0.0001
Total cholesterol (mg/dL)	215.1 (41.8)	214.9 (40.8)	215.7 (45.1)	0.4276
LDL cholesterol (mg/dL)	137.8 (38.9)	137.6 (37.7)	138.3 (42.7)	0.3977
HDL cholesterol (mg/dL)	51.5 (17.0)	50.5 (16.8)	54.8 (17.3)	< 0.0001
Triglyceride level (mg/dL)	133.0 (92.9)	137.8 (93.7)	116.3 (83.1)	< 0.0001

ARIC, Atherosclerosis Risk in Communities study; HDL, high density lipoprotein cholesterol; LDL, low density lipoprotein cholesterol

%, percent of sample. All other variable results are means

*P* values for proportions of categorical variables among Whites and African Americans were calculated using Pearson’s chi-square tests of hypothesis for independence and tests for differences between means of continuous variables. Analysis showed that all variables were statistically significant between each other except total cholesterol and LDL cholesterol

### Association between GRS or dietary patterns and MetS

The GRS was associated with MetS in both Whites and African Americans (Table 2). Each 5-increment increase in the GRS posed higher risk for MetS among African Americans (*p* < 0.001). Additionally, the highest GRS tertile had the greatest risk for MetS compared to the lowest tertile among Whites (RR = 1.28; CI: 1.23–1.33, *p* < 0.001) and notably in African Americans (RR = 1.37; CI: 1.27–1.48; *p* < 0.001).

**Table 2:** Association between a GRS or dietary patterns on metabolic syndrome over four visits in ARIC

	Risk ratio (95% confidence interval)			
	Whites	*P* value	African Americans	*P* value
	(n = 8451)		(n = 2230)	
*Genetic risk score*				
GRS range/mean (SD)	44–84/61.4 (5.3)		32–63/ 46.5 (4.2)	
GRS per 1 allele increase	**1.02 (1.02–1.03)**	** < 0.001**	**1.03 (1.02–1.04)**	** < 0.001**
GRS per 5 increment	**1.12 (1.10–1.14)**	** < 0.001**	**1.17 (1.13–1.21)**	** < 0.001**
GRS lower tertile	1.00 (ref)		1.00 (ref)	
GRS second tertile	**1.12 (1.07–1.17)**	** < 0.001**	**1.23 (1.14–1.33)**	** < 0.001**
GRS highest tertile	**1.28 (1.23–1.33)**	** < 0.001**	**1.37 (1.27–1.48)**	** < 0.001**
*Dietary pattern per 5 increment*				
No. per dietary pattern				
Western	**1.21 (1.12–1.31)**	< 0.001*	1.14 (0.97–1.33)	.102
Healthy	1.03 (0.95–1.11)	.453	1.20 (0.99–1.47)	.070
High-fat dairy	**0.72 (0.66–0.79)**	< 0.001*	**0.81 (0.69–0.96)**	.013*

Bold indicates *p* values that were significant at *p* < 0.05

^*^Bonferroni adjustment for multiple testing (*p* < 0.05/3 = 0.017)

Dietary patterns were calculated using factor analysis with the principal components factor option and the varimax rotation with correlation ≥ 0.3

MetS was regressed against the GRS adjusting for a covariate summary score composed of age, gender, sports physical activity (Baecke questionnaire), cigarette smoking status, drinker status, education level at visit 1, time in study, and 20 principle components for admixture

Dietary patterns are from Additional file 1: Table S2

Dietary pattern contents for Whites:

Western: fried foods, red meat, chips and fries, chicken with skin, processed meat, eggs, and condiments

Healthy: rice, pasta, vegetables, mashed potato, chicken without skin, lentils and beans

High-fat dairy: butter, whole milk, eggs

Dietary pattern contents for African Americans

Western: Eggs, processed meat, biscuit and cornbread, whole wheat bread, fried foods, white bread, and margarine-butter

Healthy: Chicken without skin, vegetables, lentils and beans, fruit, cooked breakfast cereal, fish, mashed potato, shellfish, and cold breakfast cereal

High-fat dairy: Butter, margarine-butter, whole milk, and cottage cheese

In longitudinal analyses, the Western dietary pattern increased MetS risk among Whites (RR = 1.21; CI: 1.12–1.31; *p* < 0.001). In contrast, the high-fat dairy pattern was associated with lower risk for MetS among Whites (RR) = 0.72; CI: 0.66–0.79; *p* < 0.001) and African Americans (RR = 0.81; CI: 0.69–0.96; *p* = 0.013); see Table 2.

### Interaction between the GRS and dietary patterns on MetS

We observed interactions with the GRS and dietary patterns (Table 3). The greatest protective effects were observed for the high-fat dairy pattern in the lowest GRS tertile among Whites (RR = 0.62; CI: 0.52–0.74; *p* < 0.001) and African Americans (RR = 0.67; CI: 0.49–0.91; *p* < 0.011). However, African Americans with the greatest burden of high-risk alleles (highest GRS tertile) and those who consumed a healthy diet, had a higher risk for MetS (RR = 1.39; CI: 1.07–1.82; *p* = 0.015). Among African Americans, the overall effect of the GRS and the Western dietary pattern increased MetS risk (RR = 7.43; CI: 1.57–35.08; *p* = 0.011). However, the confidence intervals were very wide for the point estimate and appeared unstable.

**Table 3:** Interaction between a GRS and dietary patterns on metabolic syndrome over four visits in ARIC

	Risk ratio (95% confidence interval) *P* value							
Dietary patterns	Lowest GRS Tertile	*P* value	Second GRS Tertile	*P* value	Highest GRS Tertile	*P* value	Overall effect of diet × GRS interaction	*P* value
Whites								
No. per group	3072		2487		2892		8451	
Western	1.17 (1.01–1.35)	0.031	1.28 (1.11–1.47)	0.001*	1.23 (1.09–1.38)	0.001*	1.10 (0.47–2.57)	0.831
Healthy	1.07 (0.92–1.23)	0.383	0.98 (0.85–1.13)	0.827	1.02 (0.91–1.15)	0.679	1.04 (0.45–2.41)	0.920
High-fat dairy	0.62 (0.52–0.74)	< 0.001*	0.74 (0.63–0.87)	< 0.001*	0.81 (0.71–0.91)	0.001*	0.26 (0.10–0.70)	0.008*
African Americans								
No. per group	710		795		725		2230	
Western	1.46 (1.04–2.04)	0.028	1.18 (0.93–1.50)	0.174	0.89 (0.70–1.13)	0.329	7.43 (1.57–35.08)	0.011*
Healthy	1.15 (0.73–1.81)	0.549	1.09 (0.80–1.48)	0.597	1.39 (1.07–1.82)	.015*	1.25 (0.15–10.08)	0.837
High-fat dairy	0.67 (0.49–0.91)	0.011*	0.71 (0.533–0.96)	0.024	1.03 (0.81–1.29)	0.828	0.17 (0.03–0.88)	0.035

Bold indicates *p* values that were significant at *p* < 0.05

^*^Bonferroni adjustment for multiple testing for dietary patterns (*p* = 0.05/3 = 0.017)

Dietary patterns were calculated using factor analysis with the principal components factor option and the varimax rotation with correlation ≥ 0.3

MetS was regressed against the GRS adjusting for covariate summary score composed of age, gender, sports physical activity (Baecke questionnaire), cigarette smoking status, drinker status, education level at visit 1, time in study, and 20 principle components for admixture

Dietary patterns are from Additional file 1: Table S2

Dietary pattern contents for Whites:

Western: fried foods, red meat, chips and fries, chicken with skin, processed meat, eggs, and condiments

Healthy: rice, pasta, vegetables, mashed potato, chicken without skin, lentils and beans

High-fat dairy: butter, whole milk, eggs

Dietary pattern contents for African Americans:

Western: Eggs, processed meat, biscuit and cornbread, whole wheat bread, fried foods, white bread, and margarine-butter

Healthy: Chicken without skin, vegetables, lentils and beans, fruit, cooked breakfast cereal, fish, mashed potato, shellfish, and cold breakfast cereal

High-fat dairy: Butter, margarine-butter, whole milk, cottage cheese

### Association between dietary patterns and resolution or development of MetS

**Fig. 2:**
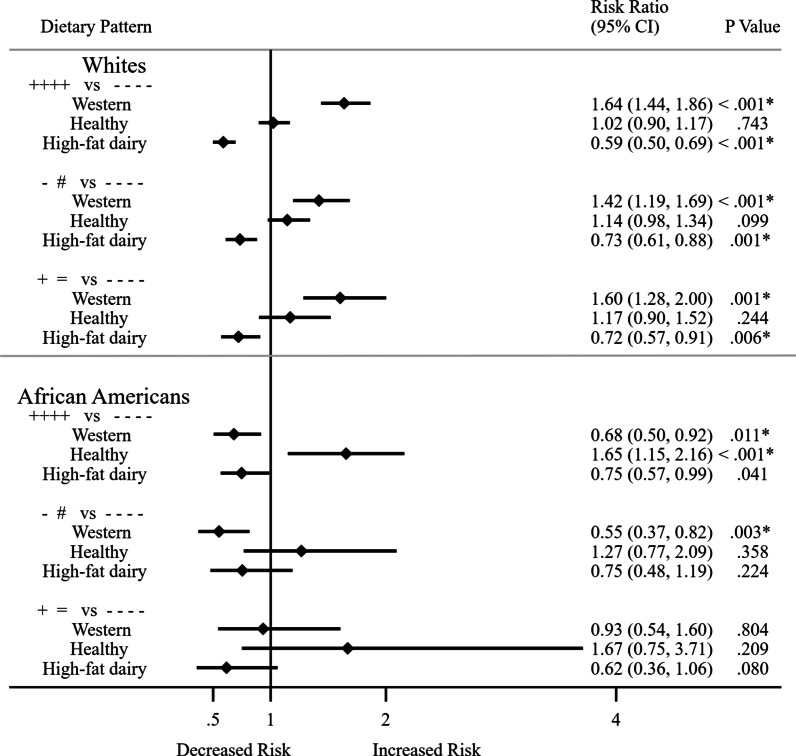
Association between dietary patterns and developing MetS or being free from metabolic syndrome among Whites and African Americans. Participants’ MetS status change was in one direction only from visits 1 to 4. *Key* +  +  +  + versus −  −  −  −: Those with MetS at all 4 visits compared with those without MetS at all 4 visits; − # versus −  −  −  −: Those free of MetS at visit 1 but developed MetS at visit 4 compared with those without MetS at all 4 visits; +  = versus −  −  −  −: Those with MetS at visit 1 but free of MetS at visit 4 compared with those without MetS at all 4 visits. Bold indicates *p* values that were significant at *p* < 0.05. Bonferroni adjustment for multiple testing for dietary patterns (*p* = 0.05/4 = 0.017). Dietary patterns were calculated using factor analysis with the principal components factor option and the varimax rotation with correlation ≥ 0.3. MetS was regressed against the GRS adjusting for a covariate summary score composed of age, gender, sports physical activity (Baecke questionnaire), cigarette smoking status, drinker status, education level at visit 1, time in study, and 20 genetic principle components for admixture. Dietary patterns are from Additional file 1: Table S2. Dietary pattern contents for Whites: Western: fried foods, red meat, chips and fries, chicken with skin, processed meat, eggs, and condiments; Healthy: rice, pasta, vegetables, mashed potato, chicken without skin, lentils and beans; High-fat dairy: butter, whole milk, eggs. Dietary pattern contents for African Americans: Western: Eggs, processed meat, biscuit and cornbread, whole wheat bread, fried foods, white bread, and margarine-butter; Healthy: Chicken without skin, vegetables, lentils and beans, fruit, cooked breakfast cereal, fish, mashed potato, shellfish, cold breakfast cereal; High-fat dairy: Butter, margarine-butter, whole milk, cottage cheese

Because MetS is comprised of individual risk factors that can improve or worsen over time, participants may have resolution or development MetS during the 11 years of follow-up. We investigated the association of dietary patterns and whether participants became free of MetS at visit 4 or developed MetS at visit 4 after having MetS at visit 1 or were free of MetS at visit 1. In these analyses, participants’ MetS status change was in one direction only from visits 1 to 4. Figure 2 and Additional file 1: Table S4 show the associations of dietary patterns with resolution or development of MetS at visit 4 by racial ancestry. Compared to those who did not have MetS at all four visits, the Western dietary pattern increased MetS risk among Whites: RR = 1.64; CI: 1.44–1.86; *p* < 0.001; n = 4499 for those with MetS at all four visits; RR = 1.42; CI: 1.19–1.69; *p* < 0.001, for those who were free of MetS at visit 1 and risk of developing MetS at visit 4 (n = 4110); and RR = 1.60; CI: 1.28–2.00; *p* = 0.001; n = 3696 for those who had MetS at visit 1 and risk of being free from MetS at visit 4.

In contrast, high-fat diary pattern consistently showed a protective association among Whites.

Compared to those who did not have MetS at all four visits, the relative risks were decreased in the following amounts: RR = 0.59; CI: 0.50–0.69; *p* < 0.001 and risk of MetS at all four visits (n = 4999); RR = 0.73; CI: 0.61–0.88; *p* = 0.001; for those who were free of MetS at visit 1 and risk of developing MetS at visit 4 (4110); RR = 0.72; CI: 0.57–0.91; *p* = 0.006; and for those who had MetS at visit 1 and risk of being free from MetS at visit 4 (n = 3696).

Among African Americans, the healthy dietary pattern was associated with higher risk for MetS (RR = 1.65; CI: 1.15–2.16; *p* ≤ 0.001; n = 1010) for those who had MetS at all four visits. The high-fat dairy patterns showed significant protective risks: RR = 0.66; CI: 0.45–0.98; *p* = 0.038; n = 1026 among those who had MetS at visit 1 and risk of being free from MetS at visit 4. However, the latter estimates did not meet the Bonferroni threshold or multiple testing (*p* < 0.017).

### Interaction between the GRS and dietary patterns and resolution or development of MetS

**Fig. 3:**
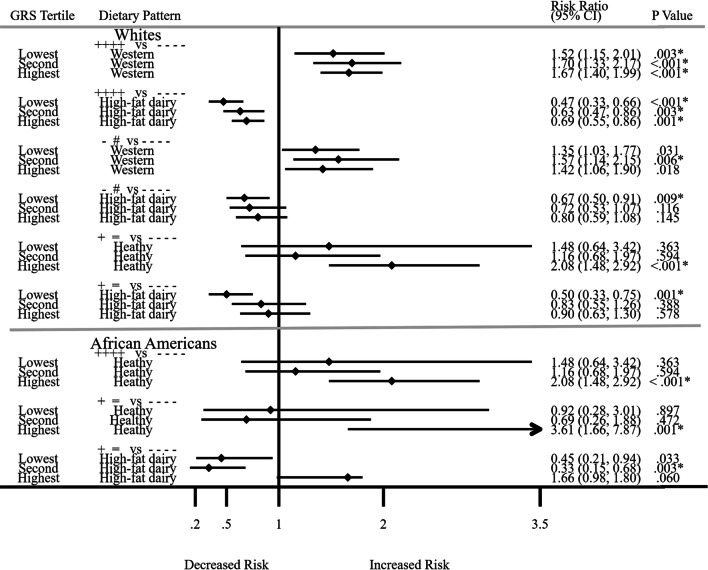
Interactions between a GRS and dietary patterns for developing MetS or being free from metabolic syndrome among Whites and African Americans. Participants’ MetS status change was in one direction only from visits 1 to 4. *Key*: +  +  +  + versus −  −  −  −: Those with MetS at all 4 visits compared with those without MetS at all 4 visits; − # versus −  −  −  −: Those free of MetS at visit 1 but developed MetS at visit 4 compared with those without MetS at all 4 visits; +  = versus −  −  −  −: Those with MetS at visit 1 but free of MetS at visit 4 compared with those without MetS at all 4 visits. Bold indicates *p* values that were significant at *p* < 0.05. Bonferroni adjustment for multiple testing for dietary patterns (*p* = 0.05/3 = 0.017). Dietary patterns were calculated using factor analysis with the principal components factor option and the varimax rotation with correlation ≥ 0.3. MetS was regressed against the GRS adjusting for a covariate summary score composed of age, gender, sports physical activity (Baecke questionnaire), cigarette smoking status, drinker status, education level at visit 1, time in study, and 20 genetic principle components for admixture

We investigated whether the GRS and dietary patterns interact to influence MetS risk beyond their independent associations. As in association analyses above, participants’ MetS status change was in one direction only from visits 1 to 4. We present results only for dietary patterns that showed significant associations with the GRS in Fig. 3. Additional file 1: Tables S5A and S5B show all significant and non-significant associations for dietary patterns and GRS tertiles by racial ancestry. We scaled dietary patterns as per 5-increment within the GRS tertiles. As in the association analysis, in all these analyses, the comparator was those who did not have MetS at any of the four visits. Among Whites who had MetS at all four visits (n = 4499), we observed higher risks for the Western dietary pattern within the GRS tertiles (*p* values for GRS tertiles 1, 2 and 3: 0.003, 0.001, 0.001, respectively). Conversely, in spite of the risk-raising GRS, the protective effects of high-fat dairy in the interaction remain among Whites for decreased risk of MetS at all four visits (*p* values for GRS tertiles 1, 2 and 3: 0.001, 0.003, 0.001, respectively; overall interaction effect: RR = 0.08; CI: 0.01–0.47; *p* = 0.005). However, the greatest protection for the high-fat dairy pattern was in the lowest GRS tertile for Whites (RR = 0.47; CI: 0.33–0.66; *p* ≤ 0.001; n = 1637) and African Americans (only significant in the lowest tertile; RR = 0.67; CI: 0.50–0.91; *p* ≤ 0.009; n = 1661) who were free of MetS at visit 1 and risk of developing MetS at visit 4.

Among African Americans who had MetS at visit 1 but were free of MetS at visit 4 compared to participants without MetS at all 4-visits, there was a protective effect of high-fat dairy intake in the second tertile (RR = 0.33; CI: 0.15–0.68); *p* = 0.003; n = 367; overall interaction effect: RR = 0.0076; CI: 0.0002–0.3372; *p* = 0.012; n = 1026). The lowest GRS tertile (RR = 0.45; CI: 0.21–0.94; *p* = 0.033; n = 358) was statistically significant but did not met Bonferroni correction for multiple testing (*p* < 0.017). Moreover, in the highest GRS tertile for those who had MetS at all four visits, the healthy dietary pattern was associated with increased MetS risk (RR = 2.08; CI: 1.48–2.92; *p* < 0.001; n = 310). In addition, there was a harmful effect of the healthy dietary pattern for those who had MetS at visit 1 and risk of being free from MetS at visit 4 (RR = 3.61; CI: 1.66–7.87; *p* < 0.001; n = 240).

### Association between dietary patterns for those who had resolution or development of MetS whose MetS status cycled between visits 1 and 4

We investigated the association of dietary patterns in participants whose MetS status cycle between visits 1 through 4. Additional file 1: Table S6 shows the associations for dietary patterns and associations with MetS among MetS status cyclers by racial ancestry. In these analyses, participants reverted back-and-forth from being free of MetS to having MetS or vice-versa from visits 1 to 4. Participants were free from or had MetS at visit 1. Among Whites, the Western dietary pattern was associated with higher risk for MetS at visit 4 (RR = 1.66; CI: 1.30–2.12; *p* < 0.001; n = 3477); as well as less likely to remain free from MetS at visit 4 (RR = 1.43; CI: 1.13–1.81; *p* = 0.003; n = 3706). However, the magnitude of effect was higher for Whites for the former association. High-fat dairy intake was associated with decreased risk of MetS in Whites (RR = 0.76; CI: 0.60–0.96; *p* = 0.019; n = 3706) and African Americans (RR = 0.66; CI: 0.45–0.96; *p* = 0.028; n = 986) and the likelihood of being free from MetS at visit 4, but this association did not meet the Bonferroni threshold cutoff of *p* < 0.017.

### Interaction between a GRS and dietary patterns for those who had resolution or development of MetS whose MetS status cycled between visits 1 and 4

We investigated the interaction between the GRS and dietary patterns in participants whose MetS status cycle between visits 1 through 4. Additional file 1: Table S7A and S7B show the interactions of a GRS and dietary pattern associations with MetS among MetS status cyclers by racial ancestry. As stated above, in these analyses, participants reverted back-and-forth from being free of MetS to having MetS or vice-versa. In these analyses we combined participants who were free from or had MetS at visit 1 to augment our sample. We observed higher risks for MetS at visit 4 among Whites in the lowest (RR = 1.77; CI: 1.19–2.74; *p* = 0.005; n = 1394) and highest (RR = 1.72; CI: 1.18–2.52; *p* = 0.005; n = 1.020) GRS tertiles for the Western pattern. Similarly, there was a less likelihood for being free from MetS at visit 4 in the highest GRS tertile for the Western dietary pattern (RR = 1.68; CI: 1.14–2.47; *p* = 0.008; n = 1094). In contrast, the high-fat dairy pattern was protective of MetS in the lowest GRS tertile and showed a decreased risk of MetS at visit 4 (RR = 0.51; CI: 0.32–0.81; *p* < 0.004; n = 1394); and for those who remained free of MetS at visit 4 (RR = 0.64; CI: 0.44–0.95; *p* = 0.027; n = 1490), but the latter association did not meet the Bonferroni threshold for multiple testing (*p* < 0.017).

Among African Americans whose MetS status cycled between visits 1 to 4, we observed the harmful effects of the Western dietary pattern was significant only in the highest GRS tertiles for those who had MetS at visit 4 (RR = 0.32; CI: 0.16–0.67; *p* = 0.002; n = 227). The high-fat dairy pattern was protective in the second GRS tertile and showed a favorable risk of being free from MetS at visit 4 (RR = 0.46; CI: 0.24–0.89; *p* = 0.20; n = 354), but the association did not meet the Bonferroni threshold for multiple testing (*p* < 0.017).

### Molecular genetic clustering pathway analysis

**Fig. 4:**
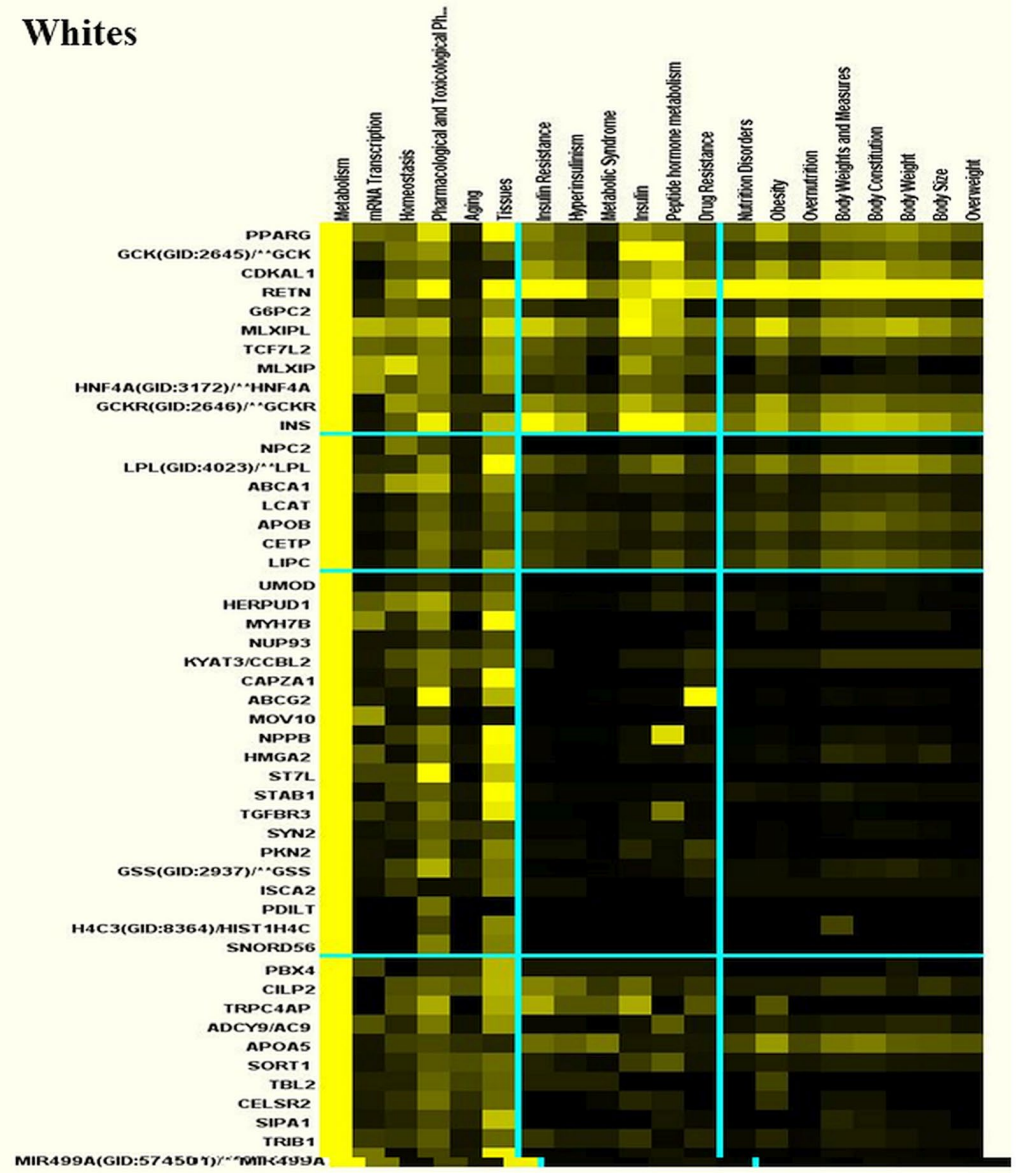
Molecular genetic clustering pathways for Whites. Molecular genetic clustering pathway analysis was performed using Literature Lab™ clustering software to find functional relationship differences among the genes by racial ancestry

**Fig. 5:**
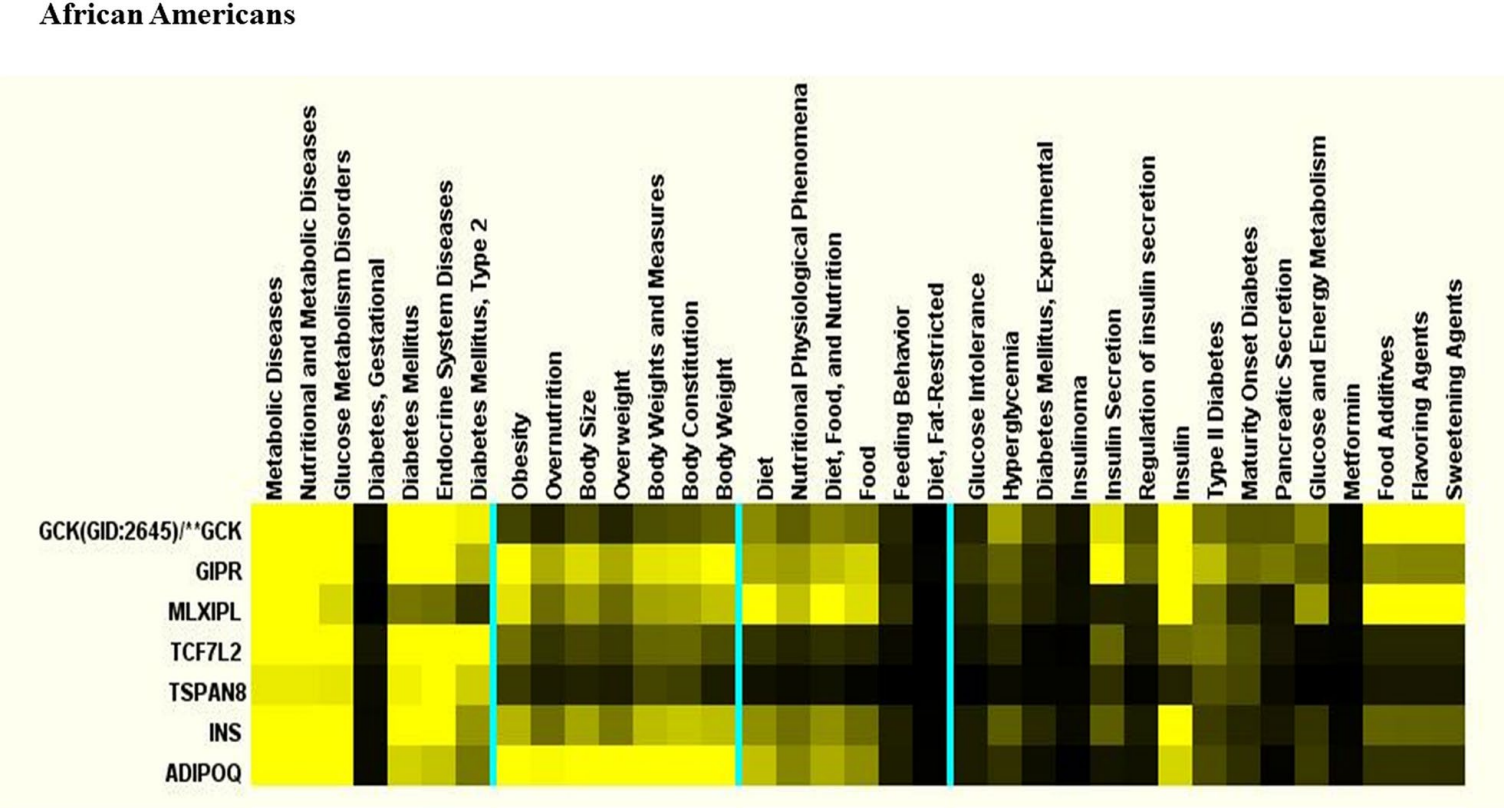
Molecular genetic clustering pathways for African Americans. Molecular clustering genetic pathway analysis was performed using Literature Lab™ clustering software to find functional relationship differences among the genes by racial ancestry

We used Literature Lab™ clustering analysis to find functional relationship differences among the genes by racial ancestry. Fewer genes but more metabolic pathways were found in African Americans than Whites (Figs. 4 and 5). In general, the top pathways for African Americans included pathways that were identified in Whites, as well as additional pathways for obesity and related body fat distribution, and lipid and carbohydrate metabolism. This may indicate that mechanisms involving gene-diet and disease risks may be more complex among African Americans than Whites.

The top five pathways by association for Whites were linoleic acid with FAD1 (26.0%) and FAD2 (67.5%), regulation of insulin secretion with insulin (88.7%), type 2 diabetes with insulin (78.5%), pancreatic secretion with insulin (70.9%), and fatty acid elongation with FAD1 (38.8%) and FAD2 (60.1%). See Table 4 for abbreviations of gene symbols and their names. When we categorized pathways by the most significant *p* values, the top pathways for Whites were CHREBP and MLXIPL (99.2%), vitamin D receptor and vitamin D (99.8%), and GCPRs-Class A Rhodopsin-Like and Rhodopsin (100%). For African Americans the top pathways by association were, CHREBP and MLXIPL (99.6%), G-Alpha signaling (99.9%) and BDNF (99.9%) with GNAS. The top five strongest pathways by most significant *p* values for African Americans were linoleic acid metabolism with FAD2 (66.7%) and FAD1 (26.8%) genes, followed by insulin secretion and type 2 diabetes with insulin pathway (86.1% and 83.1%), respectively, in addition to vitamin A and carotenoid metabolism with CY26A1 (55%) and ALDH1A2 (38.6%), and maturity onset diabetes with insulin (82.2%).

**Table 4:** Abbreviations of gene symbols and their names

Gene symbol	Gene name
ABCA1	ATP binding cassette subfamily A member 1
ABCB11	ATP binding cassette subfamily B member 11
ABCG2	ATP binding cassette subfamily G member 2 (Junior blood group)
ABO	ABO, alpha 1–3-N-acetylgalactosaminyltransferase and alpha 1–3-galactosyltransferase
AC9	Adenylate cyclase 9
ACAN	Aggrecan
ACO	Kallikrein related peptidase 15
ADIPOQ	Adiponectin, C1Q and collagen domain containing
ALDH1A2	Aldehyde dehydrogenase 1 family member A2
APOA5	Apolipoprotein A5
APOB	Apolipoprotein B
AS1	Prostaglandin D2 receptor
ATF6B	Activating transcription factor 6 beta
BAZ1B	Bromodomain adjacent to zinc finger domain 1B
BCL2	BCL2 apoptosis regulator
BCL2A1	BCL2 related protein A1
BCL7B	BAF chromatin remodeling complex subunit BCL7B
BDNF	Brain derived neurotrophic factor
CALM2	Calmodulin 2
CAPZA1	Capping actin protein of muscle Z-line subunit alpha 1
CCBL2	Kynurenine aminotransferase 3
CCK	Cholecystokinin
CDKAL1	CDK5 regulatory subunit associated protein 1 like 1
CDKN2A	Cyclin dependent kinase inhibitor 2A
CDKN2B	Cyclin dependent kinase inhibitor 2B
CELSR2	Cadherin EGF LAG seven-pass G-type receptor 2
CETP	Cholesteryl ester transfer protein
CILP2	Cartilage intermediate layer protein 2
CLCN6	Chloride voltage-gated channel 6
CLEC16A	C-type lectin domain containing 16A
CYP26A1	Cytochrome P450 family 26 subfamily A member 1
CYP26C1	Cytochrome P450 family 26 subfamily C member 1
EDEM2	ER degradation enhancing alpha-mannosidase like protein 2
Eos	Eosinophilia, familial
ERBB3	Erb-b2 receptor tyrosine kinase 3
FADS1	Fatty acid desaturase 1
FADS2	Fatty acid desaturase 2
FTO	FTO alpha-ketoglutarate dependent dioxygenase (fat Mass and obesity-associated)
G6PC2	Glucose-6-phosphatase catalytic subunit 2
GCK	Glucokinase
GCKR	Glucokinase regulator
GIPR	Gastric inhibitory polypeptide receptor
GNAS	GNAS complex locus
GSS	Glutathione synthetase
HERPUD1	Homocysteine inducible ER protein with ubiquitin like domain 1
HFE	Homeostatic iron regulator
HIGD1C	HIG1 hypoxia inducible domain family member 1C
HIST1H4C	H4 clustered histone 3
HMGA2	High mobility group AT-hook 2
HNF4A	Hepatocyte nuclear factor 4 alpha
IGF2	Insulin like growth factor 2
IGF2AS	IGF2 antisense RNA
IKZF4	IKAROS family zinc finger 4
INS	Insulin
ISCA2	Iron-sulfur cluster assembly 2
KCNQ1	Potassium voltage-gated channel subfamily Q member 1
KCNQ1OT1	KCNQ1 opposite strand/antisense transcript 1
KIAA0350	C-type lectin domain containing 16A
KLHL8	Kelch like family member 8
LCAT	Lecithin-cholesterol acyltransferase
LGR5	Leucine rich repeat containing G protein-coupled receptor 5
LINC02702	Long intergenic non-protein coding RNA 2702
LIPC	Lipase C, hepatic type
LPL	Lipoprotein lipase
HLA Class III	Caspase 7
MIR499A	microRNA 499a
MLXIP	MLX interacting protein
MLXIPL	MLX interacting protein like
MOV10	Mov10 RISC complex RNA helicase
MTHFR	Methylenetetrahydrofolate reductase
MYH7B	Myosin heavy chain 7B
NAT2	N-acetyltransferase 2
NCAN	Neurocan
NPC2	NPC intracellular cholesterol transporter 2
NPPB	Natriuretic peptide B
NUP93	Nucleoporin 93
PBX4	PBX homeobox 4
PDE3A	Phosphodiesterase 3A
PDILT	Protein disulfide isomerase like, testis expressed
PKD2	Polycystin 2, transient receptor potential cation channel
PKN2	Protein kinase N2
PPARG	Peroxisome proliferator activated receptor gamma
PRKAG2	Protein kinase AMP-activated non-catalytic subunit gamma 2
PROCR	Protein C receptor
PSRC1	Proline and serine rich coiled-coil 1
RELA	RELA proto-oncogene, NF-kB subunit
RETN	Resistin
RHBG	Rh family B glycoprotein (gene/pseudogene)
RNA5SP483	RNA, 5S ribosomal pseudogene 483
RNU6-407P	RNA, U6 small nuclear 407, pseudogene
RP11	Pre-mRNA processing factor 31
RP11-356I2.2	Long intergenic non-protein coding RNA 2539
RP4	Rhodopsin
SIPA1	Signal-induced proliferation-associated 1
SLC12A3	Solute carrier family 12 member 3
SNORD56	Small nucleolar RNA, C/D box 56
SORT1	Sortilin 1
ST7L	Suppression of tumorigenicity 7 like
STAB1	Stabilin 1
SURF6	Surfeit 6
SYN2	Synapsin II
TBL2	Transducin beta like 2
TCF7L2	Transcription factor 7 like 2
TGFBR3	Transforming growth factor beta receptor 3
TNXB	Tenascin XB
TRIB1	Tribbles pseudokinase 1
TRPC4AP	Transient receptor potential cation channel subfamily C member 4 associated protein
TSPAN8	Tetraspanin 8
UMOD	Uromodulin
VDR	Vitamin D receptor
VPS37D	VPS37D subunit of ESCRT-I

We examined similarities by association among pathways for Whites compared with African Americans and found vitamin A and carotenoid metabolism with ALDH1A2 (94.7% vs. 38.6%), ketone body regulation with insulin (89% vs. 90%), fructose/mannose metabolism with MXIPL (86.2% vs. 86.3%), glucose and energy metabolism with insulin (84.3% vs. 83.7%), and unsaturated fatty acid biosynthesis with FAD2 (55.8% vs. 55.8%) and FAD1 (44.2% vs. 44.1%) were the top pathways with a nutrition focus. We examined similarities by *p* value among Whites compared with African Americans, and found additional genes for CHREBP and MXIPL (99.2% vs. 99.6%) and linoleic acid metabolism with FAD1 (67.5% vs. 69.7%) and FAD2 (26% vs. 26.8%).

We compared differences in the pathways among racial ancestry by score, and observed that apoptosis and insulin cellular apoptosis were the top pathways. The genes associated with these pathways were: BCL2 for Whites (92.3% and 88.4%) and African Americans RELA (38% and 30%), and PPAR for Whites (20.2% and 20.9%). We looked at differences among pathways by *p* value for Whites/African Americans, and found CHREBP with MXIPL (99.2% /99.6%) and linoleic acid metabolism with FAD1 (67.5% vs. 69.7%) and FAD2 (26% vs. 26.8%) were statistically significant at *p* < 0.0001.

## Discussion

In this 11-year longitudinal study, we found that among Whites, the Western dietary pattern increased MetS risk across GRS tertiles. However, the high-fat dairy pattern was protective against MetS and its effect was strongest in the lowest GRS tertile among both racial ancestry groups. Among each racial group within GRS tertiles, the Western dietary pattern was associated with development and cycling of MetS status between visits, and the high-fat dairy pattern with being free from MetS. Fewer genes, but more metabolic pathways for obesity, body fat distribution, and lipid and carbohydrate metabolism were identified in African Americans than Whites. We found pathways for genes (FAD1/FAD2, MLXIPL) and their polymorphisms (rs174548/rs2286276, rs799165), respectively associated with a Western dietary pattern that could exacerbate MetS risk.

Higher risk for genetic traits, obesity and insulin resistance, contribute to greater risks for cardiometabolic diseases among African Americans [60]. Studies found that African Americans in general have a higher risk for type 2 diabetes and other cardiovascular diseases than Whites and greater risk for subsequent complications [60]. Our recently published study showed that African Americans have a higher prevalence of MetS compared with Whites, and that by using the 2017 AHA/ACC blood pressure guidelines, MetS prevalence has increased compared to using the previous blood pressure guidelines [52, 61].

A few studies found high-fat diary pattern to be beneficial against development of MetS [8] and type 2 diabetes [62]. Hu et al. [63] reported that the harmful Western dietary pattern was associated with development of coronary heart disease. In this study, the deleterious Western dietary pattern that was composed of fried foods, red and processed meat and sweets, increased MetS risk among Whites. Most importantly, for White participants whose MetS status cycled between visits 1 to visit 4, the Western dietary pattern and interaction with the GRS, consistently increased MetS risk.

Furthermore, within the highest GRS tertile, the healthy dietary pattern increased MetS risk among African Americans. This finding was of concern and was further investigated. Further analyses showed that the median sucrose intake (g) differed between the racial ancestry groups. Some studies show that sugar consumption increases the risk for metabolic disease [64]. High sugar intake was associated with MetS risk, type 2 diabetes, risk for overweight or obesity and cardiovascular risk factors [65, 66]. High fructose intake may be related to insulin resistance, impaired glucose tolerance, and dyslipidemia which is more prevalent among African Americans than Whites [34, 67]. Other studies show that a lower-carbohydrate higher-fat diet may be more beneficial in preventing the development of MetS and cycling of MetS status between visits [68].

Novel relationships in molecular genetic clustering pathway analysis were that of vitamin D receptor with vitamin D among Whites, CYP26A1 with vitamin A and carotenoid metabolism in African Americans, and FAD1 and FAD 2 with linoleic acid metabolism, an essential omega n-6 fatty acid, and MLXIPL with fructose/mannose metabolism among both racial ancestry groups.

Although vitamin D is known for its roles in calcium and bone metabolism, low levels of vitamin D may contribute to high fasting blood glucose, higher risk for MetS, and cardiovascular disease [69, 70]. Conversely, higher levels of vitamin D are associated with improved fasting blood glucose, reversal of insulin resistance, enhanced beta cell function, and prevention of diabetes [70, 71]. Treatment with vitamin D has been shown to abate symptoms and decrease risk of cardiometabolic diseases [69] and depression [72]. Other studies show that CY26A1 (rs4411227) was associated with vitamin A and carotenoid metabolism. Vitamin A and its’ carotenoids precursors are unsaturated fat-soluble nutrients important for vision, reproduction, immunity, cognition, and metabolism in protection against diabetes and cardiovascular diseases even among individuals with MetS who smoke, a known oxidative stressor [73, 74].

We found FAD1 and FAD2 genes were linked to rs174548, a SNP in the GRS in both Whites and African Americans. The genotype minor and major alleles can vary in different populations [75]. FAD1 and FAD2 are influential in long chain polyunsaturated acid synthesis, such as synthesis of linoleic fatty acid and arachidonic fatty acids [76, 77]. FAD1 and FAD2 genes are associated with cardiovascular diseases and other health consequences [75]. Diets rich in conjugated linoleic acid, have been shown to decrease inflammation, and consequently improve the markers of metabolic traits such as insulin sensitivity and neuropathy in diabetes [78]. Alternately, diets high in arachidonic acid are linked to pro-and inflammatory conditions that promote atherosclerotic vascular damage [75]. Farvid et al. [79] reported that higher intakes of linoleic acid, the predominant n-6 fatty acid, were associated with 13% lower risk of coronary heart disease events in a dose–response manner. The authors recommended to replace saturated fat with polyunsaturated fat for primary prevention of coronary heart disease. Habitual red meat intake was associated with higher levels arachidonic in Singapore adults [80]. Martinelli et al. [78] reported that the Western diet which carries a high ratio of arachidonic to linoleic fatty acids may be detrimental in individuals carrying the FAD genotypes linked to higher desaturase activity because they may be more prone to inflammatory conditions such as coronary artery disease and atherosclerotic damage.

In our study, MLX-interacting protein-like (MLXIPL) was linked to SNPs rs2286276 and rs799165 which were SNPs in the GRS of Whites and African Americans. In both racial ancestries we found Carbohydrate-Responsive Element-Binding Protein (CHREBP) was linked with MXIPL which was linked with fructose/mannose metabolism. Fructose as sucrose or high fructose corn syrup is associated with obesity and cardiometabolic risk factors, e.g. high levels of triglycerides, which are features of MetS [81]. Restriction of fructose could be beneficial in controlling MetS [82].

In our study, we found within GRS tertiles, harmful MetS risks were associated with the Western dietary pattern, and protective MetS risks were associated with the high-fat dairy dietary pattern. Given the interaction effects of the GRS and the Western dietary pattern that is high in saturated fat and fructose and low in linoleic fatty acid, individuals who consume a Western dietary pattern who have FAD1/ FAD2 (rs174548) and MLXIPL (rs2286276) genetic mutations may have increased risk for MetS. However, high-fat dairy intake that is high in vitamin A and vitamin D in spite of CYP26A1 (rs4411227) mutation may decrease MetS risk and potentially benefit metabolic health.

An advantage of this study is the longitudinal assessments from which we extracted covariates across four visits. ARIC is a prospective study; therefore, information bias was lessened because data on MetS components (abdominal obesity, high fasting blood glucose, elevated blood pressure, low HDL, and high triglycerides) were ascertained independently of the collection of dietary variables. In addition, there was a large number of cases of MetS at the four visits among both Whites and African Americans. Measurement bias was reduced because of the rigorous nature of the assessment protocol in the ARIC study.

A limitation of our analysis and interpretation is the relatively small sample size for African Americans, especially for the finer strata which may have contributed to type 2 error. Because of the scarcity of genetic studies performed in African Americans, we used SNPs from studies in the NHGRI catalog that were generated using predominantly European ancestry samples. African Americans appear to have more diverse complex genetic pathways than Whites. We believe that with adequate representation of SNPs genotyped in African Americans, the magnitude of effect may have been greater. Another limitation is recall bias. The FFQ was used to quantify information about a person’s intake during the previous year. Some participants may have had difficulty recalling some of the 66 food items accurately, while other foods such as traditional foods eaten by African Americans frequently, may not have been present in the FFQ. If beneficial or harmful foods were eaten frequently, but were not part of the FFQ, then it is possible that the true risk ratio is even lower or higher, respectively than we estimated.

Finally, we did not use a discovery sample population to replicate our results for the GRS due to the limitation of sample size. Neither did we used SNP beta coefficients as weights to replicate our GRS for the following reasons: Because we applied risk ratios in our analyses, we did not used odds ratios from published studies as this could have caused a more substantial bias and overestimated the risk ratios; due to MetS being a highly common disease condition found in this study in both racial ancestry populations. There were some cardiovascular studies that reported hazard ratios in the NHGRI catalog, but because MetS status can change due to improvement or worsening of risk factors, we were very skeptical to use these SNP beta coefficients which were based on risk for development of MetS.

## Conclusion

In summary, we were able to show that a GRS composed of risk-raising alleles, and the Western and high-fat dietary patterns were associated with MetS. Through interaction with the GRS, the Western dietary pattern was associated with a profoundly higher risk of development of MetS in Whites and African Americans. However, interaction with the GRS and high-fat dairy pattern was protective against MetS in both racial ancestry groups. These results were very strong for Whites, but less clear for African Americans, warranting further study in a larger sample of African Americans. Future investigations will be important to elucidate specific dietary patterns and dietary components that may confer protection against or risk for MetS based on genetic predisposition.

## Supplementary Information

**Additional file 1.** Supplemental tables.

## Data Availability

We received access to the data through the NIH controlled dbGaP repository, https://www.ncbi.nlm.nih.gov/gap with accession numbers phs000090 and phs000280. Interested researchers can request the data through this weblink.

## Abbreviations

MetS: Metabolic syndrome
GRS: Genetic risk score
AHA/ACC: American Heart Association/American College of Cardiology
RR: Risk ratios
CI: Confidence intervals
SNP: Single nucleotide polymorphisms
dbGaP: Database of Genotypes and Phenotypes
ARIC: Atherosclerosis Risk in Communities
FFQ: Food frequency questionnaire
NHGRI: National Human Genome Research Institute
GWAS: Genome-wide association studies
BMI: Body mass index
HDL: High density lipoprotein cholesterol
