# Supplementary material for: Ancestry specific associations of a genetic risk score, dietary patterns and metabolic syndrome: a longitudinal ARIC study
Source: BMC Med Genomics. 2021 May 1;14:118. doi: 10.1186/s12920-021-00961-8 (PMC8088631; doi:10.1186/s12920-021-00961-8)
Supplement: Supplementary file 1 — Additional file 1. Supplemental tables. [file 12920_2021_961_MOESM1_ESM.docx]

**Additional file 1: Table S1. Foods used in dietary pattern analysis in ARIC.**

| **Food or Food Group** | **Food Items** |
| --- | --- |
| Fried foods | Home fried or other fried foods, fish, chicken, chicken nuggets, |
| Red meat | Hamburgers, beef, pork, lamb as a sandwich or mixed dish, stew, casserole, lasagna, or in spaghetti sauce or in a main dish as a steak, roast, ham, etc. |
| Processed meat | Hot dogs, sausage, salami, bologna, etc., bacon |
| Chicken without skin | Chicken without skin |
| Chicken with skin | Chicken with skin |
| Fish | Canned tuna, dark meat fish, such as salmon, mackerel, swordfish, sardines, bluefish |
| Shellfish | Shrimp, lobster, scallops as a main dish |
| Eggs | Eggs |
| Other cheeses | Plain or part of a dish other than cottage or ricotta cheeses |
| Rice | Rice |
| Pasta | Pasta |
| Mashed potato | Mashed potato |
| Lentils and beans | Peas, lima beans, baked beans, beans or lentils-dried, cooked, canned |
| Whole wheat bread | Dark of wholegrain bread |
| White bread | White bread |
| Cooked breakfast cereal | Oatmeal, grits, cream of wheat, etc. |
| Cold breakfast cereal | Ready to eat cold breakfast cereal |
| Donuts | Donuts |
| Biscuits and cornbread | Biscuits or cornbread |
| Chips and fries | Potato chips, corn chips, French fries |
| Fruit | Fresh apples, pears, oranges, peaches, apricot, plums, bananas, canned fruit, dried fruit, grapefruit juice, orange juice, |
| Vegetables | String beans, green beans, broccoli, cabbage, cauliflower, Brussel sprouts, carrots, corn, spinach, collards or other greens, dark yellow or winter squash (e.g. acorn, butternut), sweet potatoes, tomatoes |
| Skim milk | Skim milk |
| Whole milk | Whole milk |
| Drinks non-carbonated | Drinks non-carbonated-fruit flavored punch or non-carbonated beverages, e.g. Lemonade, Punch, not diet |
| Drinks-regular | Drinks-regular- soft drinks, coke, Pepsi, 7-up, ginger ale |
| Coffee | Coffee (not decaffeinated) |
| Tea | Tea, ice, herbal, not decaffeinated |
| Margarine-butter | Margarine-butter-margarine or butter blend, pats added to food |
| Butter | Butter-pats added to food |
| Condiments | Ketchup, soy sauce, steak sauce |

Foods are those in the Atherosclerosis in Communities (ARIC) study Dietary Intake Form obtained from https://sites.cscc.unc.edu/aric/sites/default/files/public/forms/DTIA.pdf

**Additional file 1: Table S2. Dietary principal components factor scores of dietary patterns identified from food frequency questionnaire data in ARIC.**

| **Whites** |  | **African Americans** |  |
| --- | --- | --- | --- |
| (n=8,451) |  | (n=2,230) |  |
| **Food or Food Group** | **Factor Scores** | **Food or Food Group** | **Factor Scores** |
|  | **Western** |  |  |
| Fried foods | 0.69 | Eggs | 0.64 |
| Red meat | 0.61 | Processed meat | 0.55 |
| Chips and fries | 0.60 | Biscuit & cornbread | 0.45 |
| Chicken with skin | 0.50 | Cooked breakfast cereal | 0.32 |
| Processed meat | 0.43 | Fried foods | 0.35 |
| Eggs | 0.40 | White bread | 0.45 |
| Condiments | 0.30 | Margarine-butter | 0.31 |
|  | **Healthy** |  |  |
| 0- | 0.64 | Chicken without skin | 0.73 |
| Pasta | 0.60 | Vegetables | 0.73 |
| Vegetables | 0.55 | Lentils and beans | 0.63 |
| Mashed potato | 0.44 | Fruit | 0.48 |
| Chicken without skin | 0.44 | Cooked breakfast cereal | 0.45 |
| Lentils and beans | 0.34 | Fish | 0.43 |
|  |  | Mashed potato | 0.43 |
|  |  | Shellfish | 0.35 |
|  |  | Cold breakfast cereal | 0.30 |
|  | **High-fat Dairy** |  |  |
| Butter | 0.63 | Butter | 0.56 |
| Whole milk | 0.57 | Margarine-butter | 0.54 |
| Eggs | 0.32 | Whole milk | 0.52 |
|  |  | Other cheeses | 0.35 |

Dietary patterns were calculated using factor analysis with the principal components factor option, the varimax rotation, and correlation. ≥ .3.

**Additional file 1: Table S3A. SNPs chosen for inclusion in the genetic risk score for Whites.**

| **Chr** | **SNP** | **Mapped & Reported Genes** | **Functional Class** | **Phenotype** |
| --- | --- | --- | --- | --- |
| 1 | rs6668659 | NPPB | intergenic | Pulse pressure x alcohol consumption interaction |
| 1 | rs12145922 | CCBL2, PKN2, AS1 | intron | Liver enzyme levels (gamma-glutamyl transferase) |
| 1 | rs6657811 | CELSR2, SORT1 | intron | Total cholesterol, HDL cholesterol, LDL cholesterol, |
| 1 | rs1620668 | ST7L, CAPZA1, MOV10 | intron | Systolic blood pressure, diastolic blood pressure |
| 1 | rs7525133 | RHBG | Noncoding transcript exon | Visceral adipose tissue adjusted for BMI |
| 1 | rs3738441 | TGFBR3 | intron | HDL cholesterol change measurement, response to simvastatin, type II diabetes mellitus, response to fenofibrate |
| 2 | rs673548 | APOB | intron | Metabolic syndrome, lipid metabolism phenotypes, metabolic traits, metabolite levels, Total cholesterol, high density lipoprotein cholesterol levels, low density lipoprotein cholesterol levels, triglycerides |
| 2 | rs814295 | GCKR | intron | Triglyceride levels, C-reactive protein levels |
| 2 | rs1402837 | G6PC2 | intron | Hemoglobin A1c levels |
| 2 | rs552976 | G6PC2, ABCB11 | intron | Glycated hemoglobin levels |
| 3 | rs17036101 | SYN2, PPARG | intergenic | Type 2 diabetes |
| 3 | rs8192472 | CCK | intron | Body mass index |
| 3 | rs13326165 | STAB1 | intron | HDL cholesterol, Triglycerides |
| 4 | rs236996 | KLHL8 | intron | Triglyceride levels |
| 4 | rs2231142 | ABCG2, PKD2 | missense | Uric acid levels, Serum uric acid levels in response to allopurinol in gout, Urate levels in overweight individuals and lean persons, and in kidney disease |
| 6 | rs9368222 | CDKAL1 | intron | Acute insulin response, Cardiovascular disease risk factors, Body mass index, Peak insulin response, Systolic blood pressure, Pulse pressure, Fasting blood glucose |
| 6 | rs2206734 | CDKAL1 | intron | Ileal carcinoids, Body mass index, Glucose homeostasis traits |
| 6 | rs1800562 | HFE | missense | Hepcidin levels, Cardiovascular disease risk factors, cardiovascular disease risk factors, LDL cholesterol, total cholesterol, glycated hemoglobin levels, Red blood cell traits, red blood cell count, Hematological parameters, Hemoglobin, Hematocrit, Hematology traits, Iron status biomarkers (total iron binding capacity, ferritin levels, transferrin saturation, tr), Diastolic blood pressure, Iron status biomarkers, Hepcidin/ferritin ratio), Alcohol consumption (transferrin glycosylation), Mean corpuscular volume, Osteoarthritis (hip) |
| 6 | rs9295474 | CDKAL1 | intron | type 2 diabetes |
| 7 | rs1799884 | GCK | intron | Fasting blood glucose, Metabolite levels, Glycated hemoglobin levels, Glycemic traits, Hemoglobin A1c levels |
| 7 | rs2286276 | TBL2, VPS37D, MLXIPL, BAZ1B, BCL7B | Noncoding transcript exon | Hypertriglyceridemia, Total cholesterol levels |
| 7 | rs799165 | MLXIPL | intergenic | Age-related disease endophenotypes, Age-related diseases, mortality and associated endophenotypes |
| 7 | rs10224002 | PRKAG2 | intron | glomerular filtration rate, type 2 diabetes, systolic blood pressure, pulse pressure, Hemoglobin, hematocrit |
| 8 | rs1208 | NAT2 | missense | Insulin resistance/response |
| 8 | rs268 | LPL | missense | HDL cholesterol, Triglycerides, Metabolic syndrome, Medication use (HMG CoA reductase inhibitors), Medication use (HMG CoA reductase inhibitors) |
| 8 | rs301 | LPL | intron | Metabolic syndrome (bivariate traits) |
| 8 | rs17482753 | LPL | regulatory region | HDL cholesterol, Triglycerides, Lipid traits, Medication use (antithrombotic agents) |
| 8 | rs17489268 | LPL | regulatory region | Triglyceride levels, High density lipoprotein cholesterol levels |
| 8 | rs17321515 | TRIB1 | intron | Total cholesterol levels, Low density lipoprotein cholesterol levels, Triglycerides, Lipid traits |
| 8 | rs2954031 | RP11 | intron | Total cholesterol levels, High density lipoprotein cholesterol levels, Low density lipoprotein cholesterol levels, Triglycerides, Granulocyte count, Myeloid white cell count, Neutrophil count, Sum neutrophil eosinophil counts, Sum basophil neutrophil counts |
| 9 | rs2515629 | ABCA1 | intron | HDL cholesterol |
| 9 | rs3890182 | ABCA1 | intron | HDL cholesterol |
| 9 | rs2575876 | ABCA1 | intron | Total cholesterol levels, HDL cholesterol levels, LDL cholesterol levels, Lipid metabolism phenotypes, Metabolite levels (lipoprotein meCELE16Aasures), LDL cholesterol levels in current drinkers, HDL cholesterol levels in current drinkers, Triglyceride levels in current drinkers, HDL cholesterol levels x alcohol consumption (drinkers vs non-drinkers) interaction (2df), HDL cholesterol levels x alcohol consumption (regular vs non-regular drinkers) interaction (2df), Triglyceride levels x alcohol consumption (drinkers vs non-drinkers) interaction (2df), LDL cholesterol levels x alcohol consumption (regular vs non-regular drinkers) interaction (2df), Triglyceride levels x alcohol consumption (regular vs non-regular drinkers) interaction (2df), HDL cholesterol x physical activity interaction (2df test) |
| 9 | rs657152 | ABO | intron, genic upstream transcript | phytosterol, alkaline phosphatase, liver enzyme, hormone, thyroid stimulating hormone, type I diabetes mellitus, autoantibody, urinary metabolite |
| 10 | rs12243326 | TCF7L2 | intron | Two-hour glucose challenge, fasting blood insulin, Fasting blood glucose (BMI interaction) |
| 10 | rs12772424 | TCF7L2 | intron | Bipolar disorder (body mass index interaction) |
| 10 | rs7903146 | TCF7L2 | intron | physical activity measurement, body mass index, smoking behavior, fasting blood glucose, type 2 diabetes, metabolic syndrome, peripheral arterial disease, pulse pressure, insulin levels, fasting blood insulin, peak insulin response , waist circumference, hip circumference, glycosuria, A1C, physical activity, systolic blood pressure, HMG CoA reductase inhibitor use, schizophrenia, drugs used in diabetes |
| 11 | rs3741208 | INS, IGF2, IGF2-AS | noncoding transcript exon | Type 1 diabetes |
| 11 | rs10832417 | KCNQ1 | noncoding transcript exon | Blood pressure measurement (high sodium intervention) |
| 11 | rs3741378 | SIPA1, RELA | missense | Diastolic blood pressure (cigarette smoking interaction), Systolic blood pressure (cigarette smoking interaction), Diastolic blood pressure, Systolic blood pressure |
| 11 | rs2266788 | APOA5 | 3 prime UTR | Total cholesterol levels, High density lipoprotein cholesterol levels, Low density lipoprotein cholesterol levels, Triglycerides, HDL Cholesterol - Triglycerides (HDLC-TG),Triglycerides-Blood Pressure (TG-BP), Metabolic syndrome, Waist Circumference - Triglycerides (WC-TG), |
| 11 | rs174548 | FADS2, FADS1 | 5 prime UTR | eosinophil count, basophil count, blood metabolites, phospholipid delta-6 desaturase, triglycerides, total cholesterol, high density lipoprotein cholesterol, low density lipoprotein cholesterol, albumin: globulin ratio, platelet count, trans fatty acid levels, dihomo-gamma-linolenic acid, chronic kidney disease |
| 11 | rs2283228 | KCNQ1, LCAT | intron | high density lipoprotein cholesterol, type 2 diabetes |
| 11 | rs486394 | LINCO2702 | intron | metabolic syndrome |
| 12 | rs2239181 | VDR | intron | Gout |
| 12 | rs12304921 | HIGD1C | intron | Type 2 diabetes |
| 12 | rs1042725 | HMGA2 | 3 prime UTR | Height |
| 12 | rs1042725 | HMGA3 | 3 prime UTR | White blood cell count |
| 14 | rs11159086 | NPC2, ISCA2 | 3 prime UTR | Advanced glycation end-product levels |
| 15 | rs261332 | RP11 | noncoding transcript exon | Hemoglobin concentration, Total cholesterol levels, High density lipoprotein cholesterol levels, Age-related disease endophenotypes, Age-related diseases, mortality and associated endophenotypes |
| 16 | rs13333226 | UMOD | intron | Hypertension, Glomerular filtration rate in type 2 diabetes, Diastolic blood pressure |
| 16 | rs3764261 | CETP | regulatory region | Total cholesterol, HDL cholesterol, LDL cholesterol, Triglycerides, Lipid traits, HDL Cholesterol in HIV-infection, Metabolic syndrome, Metabolic syndrome (bivariate traits), Lipid metabolism phenotypes, Hematological and biochemical traits, Waist circumference and related phenotypes, Age-related macular degeneration, Exudative age-related macular degeneration, Lipoprotein-associated phospholipase A2 activity and mass, Medication use (HMG CoA reductase inhibitors) |
| 16 | rs1864163 | CETP | intron | Age-related macular degeneration, Lipid metabolism phenotypes, Total cholesterol levels, HDL cholesterol, |
| 16 | rs9939224 | CETP | intron | Metabolic syndrome (bivariate traits), High density lipoprotein cholesterol levels |
| 16 | rs1121980 | FTO | intron | high density lipoprotein cholesterol, triglycerides, waist-hip ratio, body mass index |
| 16 | rs1800775 | SLC12A3, HERPUD1, CETP, NUP93 | regulatory region | lipids, blood metabolites, total cholesterol, high density lipoprotein cholesterol, apolipoprotein A 1, coronary artery disease |
| 18 | rs6507716 | NPC1 | intron | Body mass index |
| 19 | rs1423096 | RETN | intergenic | Resistin levels |
| 19 | rs16996148 | CILP2 , PBX4 | intergenic | triglyceride, low density lipoprotein cholesterol measurement |
| 20 | rs867186 | PROCR, TRPC4AP, EDEM2, PROCR, GSS, MYH7B, MIR499A, RNU6-407P, SNORD56, RP11, RP4 | missense | Protein C levels, Anticoagulant levels, Hemostatic factors and hematological phenotypes, Diastolic blood pressure, Blood protein levels, D-dimer levels, Factor VII activity or levels, Prothrombin time, Coronary artery disease |
| 20 | rs1800961 | HNF4A | missense | Total cholesterol, HDL cholesterol, LDL cholesterol levels in current drinkers, HDL cholesterol levels in current drinkers, HDL cholesterol levels x alcohol consumption (regular vs non-regular drinkers) interaction (2df), LDL cholesterol levels x alcohol consumption (regular vs non-regular drinkers) interaction (2df), LDL cholesterol levels x alcohol consumption (drinkers vs non-drinkers) interaction (2df), Gallstone disease, HDL cholesterol x physical activity interaction (2df test)Type 2 diabetes, Type 2 diabetes (adjusted for BMI), C-reactive protein, C-reactive protein levels or total cholesterol levels (pleiotropy), Neutrophil percentage of white cells, Neutrophil count, Sum neutrophil eosinophil counts, Sum basophil neutrophil counts, Granulocyte count, Hematocrit, Myeloid white cell count, White blood cell count, Fibrinogen levels, Red blood cell count, Medication use (drugs used in diabetes), Factor VII activity |

**Abbreviations:** Chr, chromosome; SNP, single nucleotide polymorphism**.**

All SNPs were in Hardy-Weinberg (p >.05).

**Additional file 1: Table S3B. SNPs chosen for inclusion in the genetic risk score for African Americans.**

| **Chr** | **SNP** | **Mapped & Reported Genes** | **Functional Class** | **Phenotype** |
| --- | --- | --- | --- | --- |
| 1 | rs5068 | MTHFR, NPPB, CLCN6 | 3 prime UTR | Diastolic blood pressure (cigarette smoking interaction) |
| 1 | rs629301 | CELSR2, PSRC1, SORT1 | 3 prime UTR | total cholesterol, Lipids Low density lipoprotein cholesterol levels, High density lipoprotein cholesterol levels, Blood protein levels, C-reactive protein levels or total cholesterol levels, Age-related disease endophenotypes |
| 1 | rs6657811 | CELRS2, SORT1 | intron | Lipids: Total , HDL, LDL cholesterol |
| 1 | rs6668659 | NPPB | intergenic | Pulse pressure x alcohol consumption interaction |
| 1 | rs7525133 | RHBG | noncoding transcript exon | Visceral adipose tissue |
| 1 | rs3738441 | TGFBR3 | intron | HDL cholesterol change, response to simvastatin, type 2 diabetes, response to fenofibrate |
| 3 | rs182052 | ADIPOQ | intron | Adiponectin levels |
| 4 | rs236996 | AFF1 | intron | triglycerides |
| 6 | rs185819 | TNXB, HLA class III | missense | systolic blood pressure, risk-taking behavior, height |
| 7 | rs1799884 | GCK | intron | type 2 diabetes, fasting blood glucose, Hemoglobin A1c |
| 7 | rs2286276 | TBL2, VPS37D, MLXIPL, BAZ1B, BCL7B | noncoding transcript exon | triglycerides, total cholesterol |
| 8 | rs17482753 | LPL | regulatory region | Triglycerides, Medication use (antithrombotic agents), High density lipoprotein cholesterol levels, Lipid traits |
| 8 | rs17489268 | LPL | regulatory region | Triglyceride levels, High density lipoprotein cholesterol levels |
| 8 | rs301 | LPL | intron | Metabolic syndrome |
| 9 | rs1333051 | CDKN2A, CDKN2B | regulatory region | Type 2 diabetes |
| 9 | rs2777795 | ABCA1 | intron | BMI-adjusted waist-hip ratio, physical activity |
| 9 | rs651007 | ABO | intergenic | Total cholesterol, low density lipoprotein cholesterol, fasting blood glucose, serum metabolites, intercellular adhesion molecule 2, vascular endothelial growth factor receptor 2& 3, blood metabolites, e-selectin, carbohydrate sulfotransferase 15, CD109 antigen, ICAM-1, cadherin-5, factor VIII, protein jagged-1, hepatocyte growth factor, endoglin, iron status (ferritin levels), hematocrit, platelet glycoprotein 4, OX-2 membrane glycoprotein, P-Selectin, insulin levels |
| 9 | rs657152 | ABO | intergenic | thyroid stimulating hormone, interleukin-6, urinary metabolite, alkaline phosphatase, liver enzyme, type I diabetes, phytosterol levels |
| 10 | rs12243326 | TCF7L2 | intron | Fasting blood glucose, fasting insulin, 2-hour glucose |
| 10 | rs4411227 | CYP26C1, CYP26A1 | intron | Triglyceride levels |
| 11 | rs11030119 | BDNF | intron | Fat-free mass, Diastolic blood pressure, Systolic blood pressure |
| 11 | rs1004446 | INS, IGF2AS | intron | type I diabetes mellitus |
| 11 | rs174548 | FADS2, FADS1 | 5 prime UTR | blood metabolite, delta-6 desaturase, triglyceride, high density lipoprotein cholesterol, ow density lipoprotein cholesterol, total cholesterol, trans fatty acid, phospholipid , dihomo-gamma-linolenic acid, platelet count, chronic kidney disease, albumin: globulin ratio, basophil count, eosinophil count, Plasma omega-6 polyunsaturated fatty acid |
| 11 | rs2266788 | APOA5, KCNQ1 | 3 prime UTR, intron | total cholesterol, metabolic syndrome, triglycerides, total cholesterol, high density lipoprotein cholesterol, low density lipoprotein cholesterol, blood pressure, type 2 diabetes |
| 12 | rs7961581 | LGR5, TSPAN8 | intron | Type 2 diabetes |
| 12 | rs2292239 | ERBB3, IKZF4, Eos | intron | smoking, alopecia areata, type I diabetes |
| 12 | rs3764021 | CLEC2D | splice region | type I diabetes |
| 12 | rs7304841 | PDE3A, CALM2 | intron | stroke, Ischemic stroke, chronic kidney disease |
| 15 | rs261332 | LIPC, LIPC-AS1 | noncoding transcript exon | Total cholesterol levels, High density lipoprotein cholesterol levels, Age-related disease endophenotypes, Hemoglobin concentration |
| 15 | rs1879529 | ACAN | intron | BMI-adjusted waist circumference, physical activity, smoking, |
| 16 | rs16953002 | FTO | intron | Melanoma, hair color, |
| 16 | rs2292318 | LCAT | intron | HDL cholesterol levels |
| 16 | rs3764261 | CETP, HERPUD1, NUP93, SLC12A3 | regulatory region | Lipid traits, Cholesterol, total, HDL, triglycerides, Age-related macular degeneration, Metabolic syndrome, Hematological and biochemical traits, Lipoprotein-associated phospholipase A2 activity and mass, Waist circumference and related phenotypes, |
| 16 | rs12708716 | CLEC16A | intron | type I diabetes mellitus |
| 16 | rs13333226 | UMOD, PDILT | intron | glomerular filtration rate, type 2 diabetes, diastolic blood pressure, hypertension |
| 16 | rs1800775 | HERPUD1, CETP, HERPUD1 | regulatory region | blood metabolite measurement, lipids, high density lipoprotein cholesterol, total cholesterol, coronary artery disease |
| 16 | rs1864163 | CETP | intron | total cholesterol , high density lipoprotein cholesterol, lipids, age-related macular degeneration |
| 18 | rs12454712 | BCL2 | intron | Type 2 diabetes, Triglycerides, Systolic blood pressure, Body mass index, waist-to-hip ratio, Insulin Sensitivity Index, reticulocyte count, |
| 19 | rs10423928 | GIPR | intron | glucose tolerance test, obesity, systolic blood pressure, visceral adipose tissue measurement, blood urea nitrogen, body mass index |
| 19 | rs16996148 | NCAN, CILP2, PBX4 | intergenic | low density lipoprotein cholesterol, triglyceride |
| 20 | rs2057291 | GNAS, KCNQ1 | intron | Waist circumference |

**Abbreviations:** Chr, chromosome; SNP, single nucleotide polymorphism**.**

All SNPs were in Hardy-Weinberg (p >.05).

**Additional file 1: Table S4. Association between dietary patterns and resolution or development of MetS.**

|  |  | **Risk Ratio (95% Confidence Interval) P Value** | | | |  |
| --- | --- | --- | --- | --- | --- | --- |
|  |  | **Whites** |  |  | **African Americans** | |
| **Dietary patterns** | **No.** |  | **P Value** | **No.** |  | **P Value** |
| ++++ vs - - - - |  |  |  |  |  |  |
| Western | 4,499 | **1.64 (1.44-1.86)** | **< .001*** | 1,010 | **0.68 (0.51-0.91)** | **.011*** |
| Healthy | 4,499 | 1.02 (0.90-1.17) | .743 | 1,010 | **1.65 (1.22-2.25)** | **.001*** |
| High-fat dairy | 4,499 | **0.59 (0.50-0.69)** | **< .001*** | 1,010 | **0.75 (0.56-0.99)** | **.042** |
| - # vs - - - - |  |  |  |  |  |  |
| Western | 4,110 | **1.42 (1.19-1.69)** | **< .001*** | 902 | **0.55 (0.37-0.82)** | **.003*** |
| Healthy | 4,110 | 1.14 (0.98-1.34) | .099 | 902 | 1.27 (0.77-2.09) | .358 |
| High-fat dairy | 4,110 | **0.73 (0.61-0.88)** | **.001*** | 902 | 0.87 (0.61-1.23) | .423 |
| + = vs - - - - |  |  |  |  |  |  |
| Western | 3,696 | **1.60 (1.28-2.00)** | **.001*** | 1,026 | 1.02 (0.71-1.47) | .910 |
| Healthy | 3,696 | 1.17 (0.90-1.52) | .244 | 1,026 | 1.36 (0.78-2.38) | .274 |
| High-fat dairy | 3,696 | **0.72 (0.57-0.91)** | **.006*** | 1,026 | **0.66 (0.45-0.98)** | **.038** |

| ++++ vs - - - - | Those with MetS at all 4 visits vs. those without MetS at all 4 visits |  |  |
| --- | --- | --- | --- |
| - # vs - - - - | Those free of MetS at visit 1 but developed MetS by visit 4 vs. those without MetS at all 4 visits | | |
| + = vs - - - - | Those with MetS at visit 1 but free of MetS by visit 4 vs. those without MetS at all 4 visits | | |

**No. Number of participants in each dietary pattern.**

**Bold indicates p values that were significant at p < .05.**

***Bonferroni adjustment for multiple testing for dietary patterns (p=.05/3=.017).**

**Participants’ MetS status changes were in one direction only and did not reverted back and forth between visits 1 to 4.**

Dietary patterns were calculated using factor analysis with the principal components factor option and the varimax rotation with correlations ≥ .3.

MetS was regressed against the GRS adjusting for a covariate summary score composed of age, gender, sports physical activity (Baecke questionnaire), cigarette smoking status, drinker status, education level at visit 1, time in study, and 20 principle components for admixture.

**Dietary patterns are from Supplemental Table 2.**

**Dietary pattern contents for Whites:**

Western: fried foods, red meat, chips and fries, chicken with skin, processed meat, eggs, and condiments

Healthy: rice, pasta, vegetables, mashed potato, chicken without skin, lentils and beans

High-fat dairy: butter, whole milk, eggs

**Dietary pattern contents for African Americans**

Western: Eggs, processed meat, biscuit & cornbread, whole wheat bread, fried foods, white bread, and margarine-butter

Healthy: Chicken without skin, vegetables, lentils and beans, fruit, cooked breakfast cereal, fish, mashed potato, shellfish, cold breakfast cereal

High-fat dairy: Butter, margarine-butter, whole milk, cottage cheese

**Additional file 1: Table S5A. Interaction between a GRS and dietary patterns for resolution or developing MetS in Whites**

|  |  |  | **Whites** | | |  |  |  |
| --- | --- | --- | --- | --- | --- | --- | --- | --- |
|  |  |  | **Risk Ratio (95% Confidence Interval) P Value** | | |  |  |  |
| **Dietary Pattern** | **Lowest GRS Tertile** | **P Value** | **Second GRS Tertile** | **P Value** | **Highest GRS Tertile** | **P Value** | **Overall GRS x Diet Interaction** | **P Value** |
| **++++ vs - - - -** |  |  |  |  |  |  |  |  |
| No. per GRS tertile | 1,637 |  | 1,334 |  | 1,528 |  | 4,499 |  |
| Western | **1.52 (1.15-2.01)** | **.003*** | **1.70 (1.33-2.17)** | **<.001*** | **1.67 (1.40-1.99)** | **<.001*** | 1.33 (0.31-5.74) | .704 |
| Healthy | 1.08 (0.84-1.38) | .541 | 0.94 (0.72-1.21) | .615 | 1.03 (0.85-1.24) | .765 | 0.85 (0.21- 3.49) | .827 |
| High-fat dairy | **0.47 (0.33-0.66)** | **<.001*** | **0.63 (0.47-0.86)** | **.003*** | **0.69 (0.55-0.86)** | **.001*** | **0.08 (0.01-0.47)** | **.005*** |
| **- # vs - - - -** |  |  |  |  |  |  |  |  |
| No. per GRS tertile | 1,661 |  | 1,240 |  | 1,209 |  | 4,110 |  |
| Western | **1.35 (1.03-1.77)** | **.031** | **1.57 (1.14-2.15)** | **.006*** | **1.42 (1.06-1.90)** | **.018** | 1.31 (0.18-9.29) | .787 |
| Healthy | 1.11 (0.84-1.45) | .467 | 1.29 (0.97-1.72) | .083 | 1.06 (0.81-1.40) | .660 | 1.79 (0.33-9.69) | .498 |
| High-fat dairy | **0.67 (0.50-0.91)** | **.009*** | 0.75 (0.53-1.07) | .116 | 0.80 (0.59-1.08) | .145 | 0.63 (0.08-4.98) | .661 |
| **+ = vs - - - -** |  |  |  |  |  |  |  |  |
| No. per GRS tertile | 1,446 |  | 1,148 |  | 1,102 |  | 3,696 |  |
| Western | 1.49 (0.99-2.26) | .059 | **1.61 (1.13-2.29)** | **.008*** | **1.84 (1.24-2.74)** | **.003*** | 0.46 (0.03- 6.84) | .576 |
| Healthy | 1.18 (0.76-1.83) | .460 | 1.05 (0.66-1.65) | .841 | 1.28 (0.83-1.96) | .257 | 0.44 (0.02-8.96) | .597 |
| High-fat dairy | **0.50 (0.33-0.75)** | **.001*** | 0.83 (0.55-1.26) | .388 | 0.90 (0.63-1.30) | .578 | **0.052 (0.003-0.779)** | **.032** |

| **++++ vs - - - -** | Those with MetS at all 4 visits vs. those without MetS at all 4 visits |  |  |
| --- | --- | --- | --- |
| **- # vs - - - -** | Those free of MetS at visit 1 but developed MetS at visit 4 vs. those without MetS at all 4 visits | | |
| **+ = vs - - - -** | Those with MetS at visit 1 but free of MetS at visit 4 vs. those without MetS at all 4 visits | | |

**Bold indicates p values that were significant at p < .05.**

***Bonferroni adjustment for multiple testing for dietary patterns (p=.05/3=.017).**

**Participants’ MetS status changes were in one direction only and did not reverted back and forth between visits 1 to 4.**

Dietary patterns were calculated using factor analysis with the principal components factor option and the varimax rotation with correlations ≥ .3.

MetS was regressed against the GRS adjusting for a covariate summary score composed of age, gender, sports physical activity (Baecke questionnaire), cigarette smoking status, drinker status, education level at visit 1, time in study, and 20 principle components for admixture.

**Dietary patterns are from Supplemental Table 2.**

**Dietary pattern contents for Whites:**

Western: fried foods, red meat, chips and fries, chicken with skin, processed meat, eggs, and condiments

Healthy: rice, pasta, vegetables, mashed potato, chicken without skin, lentils and beans

High-fat dairy: butter, whole milk, eggs

**Additional file 1: Table S5B. Interaction between a GRS and dietary pattern for resolution or developing MetS in African Americans**

|  |  |  | **African Americans** | | |  |  |  |
| --- | --- | --- | --- | --- | --- | --- | --- | --- |
|  |  | **Risk Ratio (95% Confidence Interval) P Value** | | | | | |  |
| **Dietary Pattern** | **Lowest GRS Tertile** | **P Value** | **Second GRS Tertile** | **P Value** | **Highest GRS Tertile** | **P Value** | **Overall GRS x Diet Interaction** | **P Value** |
| **++++ vs - - - -** |  |  |  |  |  |  |  |  |
| No. per GRS tertile | 341 |  | 359 |  | 310 |  | 1,010 |  |
| Western | 0.75 (0.37-1.52) | .419 | 0.89 (0.56-1.41) | .612 | **0.46 (0.29-0.72)** | **<.001*** | 10.10 (0.42- 240.36) | .153 |
| Healthy | 1.48 (0.64-3.42) | .363 | 1.16 (0.68-1.97) | .594 | **2.08 (1.48-2.92)** | **< .001*** | 2.06 (0.04-96.58) | .712 |
| High-fat dairy | 0.70 (0.41-1.20) | .190 | 0.63 (0.38-1.05) | .076 | 1.08 (0.70-1.66) | .744 | 0.12 (0.01-2.43) | .168 |
| **- # vs - - - -** |  |  |  |  |  |  |  |  |
| No. per GRS tertile | 358 |  | 304 |  | 240 |  | 902 |  |
| Western | 0.97 (0.52-1.82) | .931 | **0.43 (0.21-0.90)** | **.026** | **0.28 (0.14-0.55)** | **<.001*** | 14.39 (0.24- 874.21) | .203 |
| Healthy | 0.92 (0.28-3.01) | .897 | 0.69 (0.26-1.88) | .472 | **3.61 (1.66-7.87)** | **.001*** | 1.15 (0.01-182.17) | .958 |
| High-fat dairy | 0.65 (0.37-1.15) | .140 | 0.71 (0.37-1.38) | .316 | 1.59 (0.97-2.61) | .068 | **0.009 (0.0002-0.3890)** | **.014*** |
| **+ = vs - - - -** |  |  |  |  |  |  |  |  |
| No. per GRS tertile | 358 |  | 367 |  | 301 |  | 1,026 |  |
| Western | 1.17 (0.57-2.42) | .667 | 1.35 (0.75-2.41) | .312 | 0.64 (0.37-1.10) | .108 | **157.85 (4.44-5618.11)** | **.005*** |
| Healthy | 0.92 (0.28-3.01) | .897 | 0.69 (0.26-1.88) | .472 | **3.61 (1.66-7.87)** | **.001*** | **0.0013 (2.50e-06-0.7011)** | **.038** |
| High-fat dairy | **0.45 (0.21-0.94)** | **.033** | **0.33 (0.15-0.68)** | **.003*** | 1.66 (0.98-2.80) | .060 | **0.00239 (0.00002-0.28079)** | **.013*** |

| **++++ vs - - - -** | Those with MetS at all 4 visits compared with those without MetS at all 4 visits |
| --- | --- |
| **- # vs - - - -** | Those free of MetS at visit 1 but developed MetS by visit 4 compared with those without MetS at all 4 visits |
| **+ = vs - - - -** | Those with MetS at visit 1 but free of MetS by visit 4 compared with those without MetS at all 4 visits |

**Bold indicates p values that were significant at p < .05.**

***Bonferroni adjustment for multiple testing for dietary patterns (p=.05/3=.017).**

**Participants’ MetS status changes were in one direction only and did not reverted back and forth between visits 1 to 4.**

Dietary patterns were calculated using factor analysis with the principal components factor option and the varimax rotation with correlations ≥ .3.

MetS was regressed against the GRS adjusting for a covariate summary score composed of age, gender, sports physical activity (Baecke questionnaire), cigarette smoking status, drinker status, education level at visit 1, time in study, and 20 principle components for admixture.

**Dietary patterns are from Supplemental Table 2.**

**Dietary pattern contents for African Americans**

Western: Eggs, processed meat, biscuit & cornbread, whole wheat bread, fried foods, white bread, and margarine-butter

Healthy: Chicken without skin, vegetables, lentils and beans, fruit, cooked breakfast cereal, fish, mashed potato, shellfish, cold breakfast cereal

High-fat dairy: Butter, margarine-butter, whole milk, cottage cheese

**Additional file 1: Table S6. Association between dietary patterns for resolution or development of MetS among those whose MetS status cycled between visits 1 to 4.**

|  |  | | **Risk Ratio (95% Confidence Interval) P Value** | | | | |
| --- | --- | --- | --- | --- | --- | --- | --- |
|  |  | **Whites** | | |  | **African Americans** |  |
| **Dietary patterns** | **n** |  | | **P Value** | **n** |  | **P Value** |
| **-/+ # vs - - - -** |  |  | |  |  |  |  |
| Western | 3,447 | **1.66 (1.30--2.12)** | | **< .001*** | 828 | 0.66 (0.42-1.05) | .077 |
| Healthy | 3,447 | 1.16 (0.92-1.47) | | .204 | 828 | 1.11 (0.62-1.99) | .720 |
| High-fat dairy | 3,447 | **0.50 (0.44-0.76)** | | **< .001*** | 828 | 0.79 (0.52-1.20) | .270 |
| **-/+ = vs - - - -** |  |  | |  |  |  |  |
| Western | 3,706 | **1.43 (1.13-1.81)** | | **.003*** | 986 | 0.99 (0.69-1.42) | .956 |
| Healthy | 3,706 | 1.18 (0.92-1.51) | | .184 | 986 | 1.23 (0.72-2.09) | .444 |
| High-fat dairy | 3,706 | **0.76 (0.60-0.96)** | | **.019** | 986 | **0.66 (0.45-0.96)** | **.028** |

| **-/+ # vs - - - -** | Those free of MetS or had MetS at visit 1 and had MetS by visit 4 compared with those without MetS at all 4 visits |
| --- | --- |
| **-/+ = vs - - - -** | Those free of MetS or had MetS at visit 1 and were free of MetS by visit 4 compared with those without MetS at all 4 visits |

**Bold indicates p values that were significant at p < .05.**

***Bonferroni adjustment for multiple testing for dietary patterns (p=.05/3=.017).**

**Participants’ MetS status changed back and forth from being free of MetS to having MetS and vice versa from visits 1 to 4.**

Dietary patterns were calculated using factor analysis with the principal components factor option and the varimax rotation with correlations ≥ .3.

MetS was regressed against the GRS adjusting for a covariate summary score composed of age, gender, sports physical activity (Baecke questionnaire), cigarette smoking status, drinker status, education level at visit 1, time in study, and 20 principle components for admixture.

**Dietary patterns are from Supplemental Table 2.**

**Dietary pattern contents for Whites:**

Western: fried foods, red meat, chips and fries, chicken with skin, processed meat, eggs, and condiments

Healthy: rice, pasta, vegetables, mashed potato, chicken without skin, lentils and beans

High-fat dairy: butter, whole milk, eggs

**Dietary pattern contents for African Americans**

Western: Eggs, processed meat, biscuit & cornbread, whole wheat bread, fried foods, white bread, and margarine-butter

Healthy: Chicken without skin, vegetables, lentils and beans, fruit, cooked breakfast cereal, fish, mashed potato, shellfish, cold breakfast cereal

High-fat dairy: Butter, margarine-butter, whole milk, cottage cheese

**Additional file 1: Table S7A. Interaction between a GRS and dietary pattern for resolution or developing MetS among Whites whose MetS status cycled between visits 1 to 4.**

|  |  |  |  | **Whites** |  |  |  |  |
| --- | --- | --- | --- | --- | --- | --- | --- | --- |
|  |  | **Risk Ratio (95% Confidence Interval) P value** | | | |  |  |  |
| **Dietary Pattern** | **Lowest GRS Tertile** | **P Value** | **Second GRS Tertile** | **P Value** | **Highest GRS Tertile** | **P Value** | **Overall GRS x Diet Interaction** | **P Value** |
| **-/+ # vs - - - -** |  | | | | | | |  |
| No. in GRS tertile | 1,394 |  | 1,033 |  | 1,020 |  | 3,447 |  |
| Western | **1.77 (1.19-2.64)** | **.005*** | 1.43 (0.88-2.32) | .145 | **1.72 (1.18-2.52)** | **.005*** | 1.64 (0.09- 28.49) | .736 |
| Healthy | 1.13 (0.77-1.65) | .533 | 1.20 (0.76-1.90) | .434 | 1.17 (0.79-1.73) | .422 | 0.73 (0.05- 10.33) | .813 |
| High-fat dairy | **0.51 (0.32-0.81)** | **.004*** | 0.62 (0.37-1.04) | .070 | 0.64 (0.41-1.02) | .061 | 1.18 (0.04-32.29) | .924 |
| **-/+ = vs - - - -** |  | | | | | | |  |
| No. in GRS tertile | 1,490 |  | 1,122 |  | 1,094 |  | 3,706 |  |
| Western | 1.31 (0.86-1.98) | .206 | 1.38 (0.9 0-2.11) | .144 | **1.68 (1.14-2.47)** | **.008*** | 0.21 (0.01-3.28) | .263 |
| Healthy | **1.49 (1.03-2.17)** | **.036** | 1.01 (0.63-1.62) | .976 | 1.02 (0.67-1.58) | .911 | 4.49 (0.30- 67.80) | .278 |
| High-fat dairy | **0.64 (0.44-0.95)** | **.027** | 0.82 (0.53-1.27) | .372 | 0.83 (0.57-1.22) | .352 | 0.15 (0.01-2.21) | .169 |

| **-/+ # vs - - - -** | Those free of MetS or had MetS at visit 1 and had MetS by visit 4 compared with those without MetS at all 4 visits |
| --- | --- |
| **-/+ = vs - - - -** | Those free of MetS or had MetS at visit 1 and were free of MetS by visit 4 compared with those without MetS at all 4 visits |

**Bold indicates p values that were significant at p < .05.**

***Bonferroni adjustment for multiple testing for dietary patterns (p=.05/3=.017).**

**Participants’ MetS status changed back and forth from being free of MetS to having MetS and vice versa from visits 1 to 4.**

Dietary patterns were calculated using factor analysis with the principal components factor option and the varimax rotation with correlations ≥ .3.

MetS was regressed against the GRS adjusting for a covariate summary score composed of age, gender, sports physical activity (Baecke questionnaire), cigarette smoking status, drinker status, education level at visit 1, time in study, and 20 principle components for admixture.

**Dietary patterns are from Supplemental Table 2.**

**Dietary pattern contents for Whites:**

Western: fried foods, red meat, chips and fries, chicken with skin, processed meat, eggs, and condiments

Healthy: rice, pasta, vegetables, mashed potato, chicken without skin, lentils and beans

High-fat dairy: butter, whole milk, eggs

**Additional file 1: Table S7B. Interaction between a GRS and dietary pattern for resolution or developing MetS among African Americans whose MetS status cycled between visits 1 to 4.**

|  |  |  | **African Americans** | | |  |  |  |
| --- | --- | --- | --- | --- | --- | --- | --- | --- |
|  |  |  | **Risk Ratio (95% Confidence Interval) P Value** | | | |  |  |
| **Dietary Pattern** | **Lowest GRS Tertile** | **P Value** | **Second GRS Tertile** | **P Value** | **Highest GRS Tertile** | **P Value** | **Overall GRS x Diet Interaction** | **P Value** |
| **/+ # vs - - - -** |  |  |  |  |  |  |  |  |
| No. in tertile | 324 |  | 277 |  | 227 |  | 828 |  |
| Western | 1.16 (0.57-2.37) | .676 | 0.62 (0.24-1.56) | .308 | **0.32 (0.16-0.67)** | **.002*** | **139.97 (1.18-16661.66)** | **.043** |
| Healthy | 1.55 (0.74-3.24) | .240 | 0.77 (0.22-2.62) | .672 | 1.12 (0.39-3.27) | .832 | 1.778 (0.004-876.779) | .856 |
| High-fat dairy | 0.87 (0.48-1.56) | .634 | **0.34 (0.15-0.77)** | **.010*** | 1.55 (0.92-2.60) | .102 | 0.052 (0.001-2.79) | .146 |
| **-/+ = vs - - -** |  |  |  |  |  |  |  |  |
| No. in tertile | 372 |  | 354 |  | 260 |  | 986 |  |
| Western | 1.19 (0.63-2.23) | .591 | 1.50 (0.88-2.55) | .138 | **0.39 (0.20-0.77)** | **.007*** | 15.21 (0.45-511.27) | .129 |
| Healthy | 1.63 (0.62-4.27) | .321 | 0.74 (0.29-1.85) | .516 | 2.03 (0.89-4.61) | .092 | 0.31 (0.0005-195.36) | .725 |
| High-fat dairy | 0.55 (0.29-1.07) | .080 | **0.46 (0.24-0.89)** | **.020** | 1.36 (0.68-2.73) | .385 | 0.0179 (0.0002-1.9364) | .092 |

| **-/+ # vs - - - -** | Those free of MetS or had MetS at visit 1 and had MetS by visit 4 compared with those without MetS at all 4 visits |
| --- | --- |
| **-/+ = vs - - - -** | Those free of MetS or had MetS at visit 1 and were free of MetS by visit 4 compared with those without MetS at all 4 visits |

**Bold indicates p values that were significant at p < .05.**

***Bonferroni adjustment for multiple testing for dietary patterns (p=.05/3=.017).**

**Participants’ MetS status changed back and forth from being free of MetS to having MetS and vice versa from visits 1 to 4.**

Dietary patterns were calculated using factor analysis with the principal components factor option and the varimax rotation with correlations ≥ .3.

MetS was regressed against the GRS adjusting for a covariate summary score composed of age, gender, sports physical activity (Baecke questionnaire), cigarette smoking status, drinker status, education level at visit 1, time in study, and 20 principle components for admixture.

**Dietary patterns are from Supplemental Table 2.**

**Dietary pattern contents for African Americans**

Western: Eggs, processed meat, biscuit & cornbread, whole wheat bread, fried foods, white bread, and margarine-butter

Healthy: Chicken without skin, vegetables, lentils and beans, fruit, cooked breakfast cereal, fish, mashed potato, shellfish, cold breakfast cereal

High-fat dairy: Butter, margarine-butter, whole milk, cottage cheese
